# Desiccation-induced viable but nonculturable state in *Pseudomonas putida* KT2440, a survival strategy

**DOI:** 10.1371/journal.pone.0219554

**Published:** 2019-07-19

**Authors:** Laura Abisaí Pazos-Rojas, Ligia Catalina Muñoz-Arenas, Osvaldo Rodríguez-Andrade, Lesther Emanuel López-Cruz, Orestes López-Ortega, Fábio Lopes-Olivares, Silvia Luna-Suarez, Antonino Baez, Yolanda Elizabeth Morales-García, Verónica Quintero-Hernández, Miguel Angel Villalobos-López, Jesús De la Torre, Jesús Muñoz-Rojas

**Affiliations:** 1 Ecology and Survival of Microorganisms Research Group (ESMRG), Laboratorio de Ecología Molecular Microbiana (LEMM), Centro de Investigaciones en Ciencias Microbiológicas (CICM), Instituto de Ciencias (IC), Benemérita Universidad Autónoma de Puebla (BUAP), Puebla, Mexico; 2 Centro de Investigación en Biotecnología Aplicada, Instituto Politécnico Nacional, Tepetitla, Tlaxcala, Mexico; 3 Licenciatura en Biotecnología, Facultad de Ciencias Biológicas, BUAP, Puebla, Mexico; 4 Facultad de Ingeniería Ambiental, Universidad Popular Autónoma de Puebla, Puebla, Mexico; 5 Inserm U932, Institute Curie, Paris, France; 6 Núcleo de Desenvolvimento de Insumos Biológicos para a Agricultura (NUDIBA), Universidade Estadual do Norte Fluminense Darcy Ribeiro (UENF), Rio de Janeiro, Brazil; 7 CONACYT, ESMRG, LEMM, CICM, IC, BUAP, Puebla, México; 8 Department of Environmental Protection, CSIC-Estación Experimental del Zaidín, Granada, Spain; Dong-A University, REPUBLIC OF KOREA

## Abstract

The potential of *Pseudomonas putida* KT2440 to act as a plant-growth promoter or as a bioremediator of toxic compounds can be affected by desiccation. In the present work, the bacterial survival ratio (BSR) in response to air desiccation was evaluated for *P*. *putida* KT2440 in the presence of different protectors. The BSR in the presence of nonreducing disaccharides, such as trehalose, was high after 15 days of desiccation stress (occurring at 30°C and 50% relative humidity), whereas in the absence of a protector the bacterial counts diminished to nondetectable numbers (ca 2.8 log CFU/mL). The LIVE/DEAD staining method showed that bacteria protected with trehalose maintained increased numbers of green cells after desiccation while cells without protection were all observed to be red. This indicated that nonprotected bacteria had compromised membrane integrity. However, when nonprotected bacteria subjected to 18 days of desiccation stress were rehydrated for a short time with maize root exudates or for 48 h with water (prolonged rehydration), the bacterial counts were as high as that observed for those not subjected to desiccation stress, suggesting that the cells entered the viable but nonculturable (VBNC) state under desiccation and that they returned to a culturable state after those means of rehydration. Interestingly an increase in the green color intensity of cells that returned to a culturable state was observed using LIVE/DEAD staining method, indicating an improvement in their membrane integrity. Cellular activity in the VBNC state was determined. A GFP-tagged *P*. *putida* strain expressing GFP constitutively was subjected to desiccation. After 12 days of desiccation, the GFP-tagged strain lost culturability, but it exhibited active GFP expression, which in turn made the cells green. Furthermore, the expression of 16S rRNA, *rpoN* (housekeeping), *mutL*, *mutS* (encoding proteins from the mismatch repair complex), and *oprH* (encoding an outer membrane protein) were examined by RT-PCR. All evaluated genes were expressed by both types of cells, culturable and nonculturable, indicating active molecular processes during the VBNC state.

## Introduction

*Pseudomonas putida* KT2440 is a nonpathogenic gram-negative bacterium that is able to colonize the rhizosphere of several plants [1], degrade aromatic compounds [2,3], and promote the growth and health of plants [4–6]. *P*. *putida* KT2440 has been widely used as a model in biodegradation and environmental adaptation studies [7,8], showing complex chemosensory systems, signal transduction, genetic regulation, and environmental stress responses that explain its high metabolic and adaptive versatility [7,9–11]. Despite this versatility, the survival of *P*. *putida* KT2440 decreases drastically after the loss of water, as documented in studies of freeze-drying and drying, both under vacuum conditions [12,13]. The potential of *P*. *putida* KT2440 for use in the bioremediation of soils and plant growth promotion could be affected by drought, temperature and pH fluctuations, high salinity, low nutrient availability, and desiccation. These conditions are limiting factors that determine the survival of all bacteria [14–17]. In particular, desiccation is a highly restrictive factor in regard to the development of any organism, including bacteria [17–19]. Some bacteria are highly tolerant to desiccation, such as *Enterobacter* sp. UAPS03001, *Klebsiella variicola* T29A and *Paraburkholderia unamae* MTl-641, but others are very sensitive to desiccation stress, such as *Bradyrhizobium japonicum* USDA 110 and *Burkholderia sacchari* LMG 19450 [6,20,21]. Under water-limited conditions, tolerance to desiccation is fundamental for any bacterial species associated with seeds to maintain their plant-growth promoting features, which normally recover after rehydration [20]. The bacteria that are best adapted to desiccation-rehydration processes will be the most competitive in environments with low water availability; for example, tolerant bacteria that were adhered to seeds and desiccated for 18 days were rehydrated and showed good root colonization during plant development [6]. In addition, bacteria tolerant to desiccation have the capability to rapidly resume activity and show increased transcription levels when water becomes available again [22].

The presence of stressors such as UV radiation, heavy metals, nutrient limitation, low temperatures, salinity, desiccation and oxidation can lead to a viable but nonculturable (VBNC) state [23–26], in which bacteria remain viable and metabolically active but fail to grow on standard culture media [27,28]. Cells suffer metabolic changes, such as a reduction in nutrient transport, respiration rates, and macromolecular synthesis, during the VBNC state [29]. Furthermore, a continuous gene expression occurs in cells in VBNC state [30], which has been proposed to be definitive proof that cells remain metabolically active and are not dead [23]. In some cases, the removal of the inducing stressors and/or the provision of suitable conditions for VBNC cells can restore their ability to grow and therefore their culturability [31,32]. At least eighty-five bacterial species have been reported to enter the VBNC state, most of them pathogenic species [32], but the behavior of beneficial plant-associated microorganisms remains to be fully investigated.

Bacterial desiccation under vacuum conditions is completely different from that occurring in natural environments because desiccation in the environment occurs without negative pressure. Air desiccation studies could better represent the level of tolerance of microorganisms, and their beneficial potential could be affected by water limitation. Therefore, in the present work, the capability of *P*. *putida* KT2440 to tolerate air desiccation was studied, and its entrance into a VBNC state as a survival strategy is discussed.

## Materials and methods

### Bacterial growth and desiccation assays

*P*. *putida* KT2440 cells were grown until the stationary phase in LB (Luria-Bertani) liquid medium supplemented with 100 μg/mL chloramphenicol (LB-Cm^100^) [12]. Desiccation assays were conducted as previously described [33]. Three hundred and sixty milliliters of bacterial growth suspension was subdivided into aliquots of 15 mL. Each aliquot was centrifuged at 5000 rpm for 10 min, the supernatant was removed and the pellet was resuspended in the same volume of sterile distilled water. This process was repeated twice but in the second round the bacterial pellet in each tube was resuspended with 200 mM of different protectors. A control using only water was included. Each suspension was aliquoted in microtubes of 1.5 mL capacity with 500 μL of bacterial suspension and covered with sterile cotton. Five samples from each treatment were used to determine the bacterial density (Colony Forming Units (CFU)/mL) contained in the suspensions before desiccation using the Massive Stamping Drop Plate (MSDP) method [34–36]. Desiccation was carried out at 30°C and 50% relative humidity (RH). The bacterial density (CFU/mL) was monitored every 3 days after the beginning of desiccation (DABD) by taking 5 samples and rehydrating them with water (500 μl) for 20 min. The medium used for bacterial quantification was LB agar (1.5% W/V)-Cm^100^. The BSR to air desiccation was calculated as the ratio of the log of the number of bacterial cells present in the suspension at any time post desiccation (PD) plus one to the log number of viable cells before desiccation (BD), all multiplied by 100; BSR = [(logPD + 1)/logBD] × 100 [6,12,20]. A BSR value of 100 indicates that all bacteria survived after desiccation stress, while a BSR value of 0 indicates that no bacteria survived. Samples were weighed both before desiccation and after desiccation to calculate the water lost from each sample. All samples attained complete desiccation at 5 DABD.

### Adherence to maize sprouts and colonization of *P*. *putida* KT2440 after desiccation

Cells of *P*. *putida* KT2440 were desiccated with or without a protector (trehalose 200 mM) following the methodology described above. At 18 DABD, a total of 25 dried samples with and without the protector were rehydrated with water (500 μL) for 20 min and transferred to a Falcon 15 mL centrifuge tube. Axenic maize sprouts were obtained according to Morales-García *et al*. (2011) [37] and then inoculated by submerging the germinate in the bacterial suspension for 1 h. Two control treatments were included, the first consisting of 20 sprouting seeds submerged in water for 1 h and the second consisting of 20 sprouting seeds submerged in 200 mM trehalose. Five sprouts from each treatment were used to determine the number of bacteria adhered to the maize germinates using the MSDP method [6,20,37]. The remaining sprouts were transferred to 50 mL Falcon centrifuge tubes containing 6.4 g of sterile vermiculite amended with 25 mL of sterile water, and the tubes were placed in a plant growth chamber for 15 days at 25°C and 80% RH with a photoperiod of 16 h light/8 h darkness. Every 3 days, the plants were irrigated to maintain substrate moisture. Rhizosphere samples were collected to evaluate colonization as previously described using five replicate plants from each treatment [20,36,38]. For this, the vermiculite adhered to the roots and considered as the rhizosphere was resuspended in water at a ratio of 1:10 (W/V). This suspension was vortexed for 3 min, and the resulting suspension was serially diluted. The bacterial abundance was determined according to the MSDP method using LB-agar (1.5% W/V)-Cm^100^. In addition to colonization, the bacterial membrane integrity was evaluated for each treatment (with and without trehalose) at each stage of bacterial establishment. Thus, suspensions of the bacteria before desiccation, at 18 DABD and rehydrated for 20 min, bacterial suspensions from sprouting seeds and from the rhizosphere were assayed. For all the evaluated samples, the MFI (mean of fluorescence intensity) values were calculated. Both the membrane integrity assays and MFI measurements are described in the section “Membrane damage to *P*. *putida* KT2440 during desiccation”.

### Rehydration of *P*. *putida* KT2440 with maize root exudates

Two types of root exudates were obtained: 1) Root exudates from the early stages of growth. For this treatment, five mL of water was used to wash an agar-water plate in which 10 axenic seeds had previously germinated [37]. The collected exudates were stored at -20°C until they were used. 2) Root exudates from plants grown for 12 days. This type of root exudate was obtained from hydroponic axenic systems. Each system consisted of a 300 mL flask containing 100 mL of liquid MS-J medium (Morales-García *et al*., 2011). A metallic ring was placed inside each flask in contact with the medium, and the systems were covered with cotton. The hydroponic systems were sterilized before use. A previously germinated axenic maize sprout was placed in each ring such that the root remained in contact with the MS-J medium, and the root exudates were allowed to accumulate within the medium. The plant growth conditions were 25°C and 80% RH with a photoperiod of 16 h light/8 h darkness. The root exudates were collected after 12 days of plant growth and were stored at -20°C until they were used.

Both types of root exudates were used to rehydrate desiccated cells of *P*. *putida* KT2440. Desiccated bacterial cells were obtained as described before, and 5 samples were prepared for each treatment. The bacterial abundances in the suspensions before desiccation and at 18 DABD were determined using the MSDP method with five independent samples for each treatment. The desiccated cells were rehydrated for 20 min and 3, 6, 9, 12, 24, 27, 30 and 48 h with the plant root exudates. Cells rehydrated only with water were used as controls.

### Ability of *P*. *putida* KT2440 to grow in the presence of maize root exudates under static conditions

To test whether *P*. *putida* KT2440 can use maize root exudates as a carbon source and grow under static conditions, similar to the procedure used in the rehydration experiments, bacterial cells were grown until the stationary phase in liquid LB-Cm^100^ medium (two 50 mL flasks containing 15 mL of culture). The bacterial suspensions were washed and resuspended with the same volume of maize root exudates and water as a control. The suspensions were serially diluted (1:10) to quantify the number of bacteria. For the exudate treatments, the bacterial dilutions were performed using the exudates. All dilution tubes were kept at room temperature under static conditions for 24 and 48 h. The bacterial abundance in each dilution was determined at those experimental times.

### Membrane damage to *P*. *putida* KT2440 during desiccation

Bacterial membrane damage was evaluated using the L7007 LIVE/DEAD BacLight Bacterial Viability Kit for microscopy (Molecular Probes Invitrogen Detection Technologies) and the observation of stained cells using fluorescence microscopy. This kit uses SYTO 9 and propidium iodide to discriminate between live cells with intact membranes (green fluorescence) and dead cells with compromised membranes (red fluorescence). Cells of *P*. *putida* KT2240 were grown until the stationary phase, and samples of washed bacterial suspension were desiccated for 18 days following the methodology described previously. Membrane integrity was tested in 5 samples before desiccation, and in five samples from 3, 6, 9, 12, 15 and 18 DABD rehydrated for 20 min, and the bacterial abundances were determined to calculate the BSR values. The cells at 18 DABD were also rehydrated for 24 and 48 h to determine the bacterial abundance and membrane integrity. The samples were observed with the VE-146YT fluorescence microscope at 100× using G (excitation 500–550 nm, red bacteria) and B filters (excitation 420–490 nm, green bacteria) following the supplier’s instructions. The bacterial images were examined by means of fluorescence intensity (MFI) graphs to evaluate the distribution of propidium iodide and SYTO 9 alone or in combination (MERGE); the analysis was performed along a line randomly traced through the cells. Pixel intensity information per fluorescence channel was extracted with ImageJ (v1.43u, NIH, USA, public domain). Each graph was generated from a minimum of fifteen randomly selected bacteria for each condition per experiment using Microsoft Excel (v14.3.9, Microsoft Corporation, Redmond, WA, USA). The MFI data were examined sequentially by ImageJ and graphed using Prism software.

### TEM analysis of *P*. *putida* KT2440 under desiccation

TEM analysis was performed with the BD samples and those from 6, 12 and 18 DABD. The methodology used to prepare the samples was carried out in four stages: 1) fixation of the sample with a mixture of 2.5% glutaraldehyde, 4% paraformaldehyde and 0.05 M phosphate buffer; 2) sample washing with phosphate buffer; 3) sample dehydration with alcohol at different concentrations (15, 30, 50, 70, 90 and 100%), starting the wash from the lowest to the highest concentration of alcohol; and 4) inclusion of the sample in LR White (Sigma) starting with a dilution of 2:1 (alcohol:resin) for 6 h at 4°C. Subsequently, the solution was discarded, and a dilution of 1:1 (alcohol:resin) was added under the same conditions. Finally, the solution was discarded, pure resin was added for 24 h at 4°C (two times) and the samples were allowed to polymerize at 60°C for 24 h. After inclusion, the samples were microtomized for the analysis of contrast, and the samples were observed with TEM.

### Desiccation of *P*. *putida* KT2440 tagged with GFP

GFP-tagged *P*. *putida* KT2440, generated by the site-specific insertion of miniTn7-*gfp* at an extragenic location near *glmS*, which constitutively expresses the *gfp* gene [39], was used in this work. The GFP-tagged bacterial strain was subjected to desiccation for 18 days following the methodology described above. The bacterial abundance was determined at 3, 6, 9, 12, 15 and 18 DABD after rehydration, and BSR values were calculated. The microscopic examination of GFP fluorescence using a Zeiss Axioplan ZE155 microscope (Germany) (filters BP546/ FT580/ LP590) was performed with the same samples used to quantify the bacterial abundance, and the fluorescence intensity was measured with a HIDEX Chameleon Multilabel Detection Platform multiwell plate reader at an excitation wavelength of 395 nm and emission wavelength of 509 nm. The samples were observed at 100×.

### Expression of some constitutive genes of *P*. *putida* KT2440 under desiccation

Total RNA was extracted from *P*. *putida* KT2440 cells before desiccation, and from 20 min-rehydrated cells (from samples at 6, 12 and 18 DABD). RNA extraction was performed using the hot acidic phenol-chloroform method (3 replicates of 1.5 mL) [40]. Cells were lysed using 0.5% SDS, 20 mM sodium acetate and 10 mM EDTA, and RNA was extracted twice with hot acid phenol:chloroform followed by two extractions with phenol:chloroform isoamyl alcohol. Total RNA was precipitated with absolute ethanol and then washed in 70% ethanol (molecular grade). Finally, the RNA sample was resuspended in DEPC water and stored at -80°C until use. To reduce genomic DNA contamination, the RNA isolated from each sample was treated with the Invitrogen TURBO DNA-free Kit. RNA integrity was evaluated in 2% agarose gel, and its concentration was measured using a Thermo Scientific NanoDrop 2000/2000c spectrophotometer. The cDNA of each gene for which expression was evaluated was obtained using the High-Capacity cDNA Reverse Transcription Kit from Applied Biosystems, with 2 μg RNA as the template and 10 μM of specific DNA primers (antisense primers, Table 1). Retrotranscription reactions were performed at 25°C per 10 min, 37°C per 120 min, 85°C per 5 min, and 4°C per 10 min. Total cDNA was quantified using a Thermo Scientific NanoDrop 2000/2000c spectrophotometer. To amplify each gene of interest from the cDNA obtained, PCRs were performed. The PCR conditions were 1 cycle for 5 min at 95°C, 25 cycles at 95°C for 30 seconds, 63°C for 30 seconds, and 72°C for 20 seconds, 1 cycle at 72°C for 8 min, and a final step of 10 min at 4°C. The amplified products were visualized by electrophoresis in 1% agarose gel (30 min, 100 volts) using GelRed Nucleic Acid Gel Stain (Biotium).

**Table 1:** Oligonucleotides used for the analysis by RT-PCR and RT-qPCR.

Gene	Product	Forward (5’→3’)	Reverse (5’→3’)
*oprH*	Outer membrane porine	GCCGCTACTACATGACCTATG	CCGAACAGCTTGGTGGTAT
*mutL*	DNA mismatch repair system	GCAGCTCAAGGGTATCTACATC	CTTGAGGCGCTCGTACATTAT
*mutS*	DNA mismatch repair system	CACCCACTACTTCGAACTGAC	GGAACACGATGCGTTCATTG
16S RNA	16S RNA	TGTGAAGAAGGTCTTCGGATTG	CAGAGTTAGCCGGTGCTTATT
*rpoN*	Sigma factor σ54	CTGGTAGAACTGAACCAGGAAG	GTTGCGCATGAAGGTGTTG

Real-time qPCR was performed using an Applied Biosystems 7500 Fast Real Time PCR System. Reactions were performed by using SYBR Green PCR Master Mix as a signal reporter and 30 amplification cycles. Each reaction was composed of 10 ng of cDNA and 6 μM of sense and antisense primers in a total volume of 20 μL. RT-qPCR was performed in 96-well microtiter PCR plates using the following amplification conditions: 1 cycle of 5 min at 95°C and 30 two-step cycles at 95°C for 30 seconds and 63°C for 30 seconds. Each reaction was performed in triplicate. Data were analyzed using the 2^-ΔΔC^_T_ method [41]. In our work, ΔΔC_T_ = [(C_T_ gene of interest–C_T_ internal control) AD–(C_T_ gene of interest–C_T_ internal control) BD], where AD means after desiccation and BD means before desiccation. The expression of the *rpoN* gene was used as an endogenous control to normalize the amount of mRNA obtained from a target gene. The expression data obtained for each time point were normalized to the expression of each gene obtained before desiccation.

## Results

### Survival of *P*. *putida* KT2440 under air desiccation (30°C and 50% RH) was increased by nonreducing disaccharides

The tolerance of *P*. *putida* KT2440 to air desiccation was explored in the presence or absence of 200 mM of diverse substances used as possible protectors (Table 2). *P*. *putida* KT2440 was sensitive to desiccation, and bacteria were not detected at 12 DABD after rehydration when no protector was added. Some of the explored compounds protected this bacterium from desiccation stress (Table 2). The best protectors were the nonreducing disaccharides (trehalose and sucrose) and the monosaccharide glucose, followed by fructose and some polyalcohols (dulcitol, mannitol and myo-inositol). Other compounds were less successful in protecting this bacterium, such as maltose, lactose and adonitol. Galactose was unable to protect *P*. *putida* KT2440 from desiccation, and the BSR was similar to that observed without protector addition. The presence of amino acids had a negative effect on bacterial survival under the evaluated desiccation conditions in comparison with that in the absence of a protector.

**Table 2:** BSR of *P*. *putida* KT2440 cells subjected to air desiccation in the presence or absence of protectors (200 mM).

	0 DABD	3 DABD	6 DABD	9 DABD	12 DABD	15 DABD
Trehalose	100	100.79 (± 2.75)	86.66 (± 3.83)	82.89 (± 1.5)	78.65 (± 4.41)	79.56 (± 2.79)
Sucrose	100	96.67 (±1.88)	88.77 (± 1.11)	75.99 (± 6.61)	77.05 (± 5.10)	79.05 (± 5.10)
Glucose	100	84.78 (± 1.32)	85.62 (± 1.13)	84.36 (± 2.55)	84.13 (± 1.19)	80.84 (± 0.82)
fructose	100	74.62 (± 3.92)	73.83 (± 1.51)	73.12 (± 4.74)	71.05 (± 4.59)	65.793 (± 6.05)
Dulcitol	100	97.65 (± 0.58)	80.00 (± 2.35)	68.89 (± 8.08)	63.65 (± 4.31)	55.31 (± 9.01)
Mannitol	100	97.34 (± 1.15)	80.33 (± 0.69)	68.61 (± 2.61)	53.59 (± 5.54)	50.26 (± 6.98)
Myo-Inositol	100	99.57 (± 0.94)	80.76 (± 3.83)	74.92 (± 3.32)	55.70 (± 6.93)	47.56 (± 4.3)
Raffinose	100	78.86 (± 8.39)	69.37 (± 4.32)	55.43 (± 1.11)	54.10(± 3.79)	47.54 (± 3.79)
Sodium gluconate	100	87.41 (± 4.25)	52.04 (± 4.09)	42.34 (± 5.16)	44.20 (± 5.56)	35.15 (± 5.46)
Arabinose	100	58.13 (± 10.51)	26.91 (± 8.91)	36.60 (± 9.40)	17.78 (± 8.40)	20.06 (± 8.86)
Maltose	100	92.00 (± 3.25)	14.36 (± 14.37)	20.76 (± 4.82)	15.73 (± 4.81)	9.99 (± 5.33)
Xylose	100	70.34 (± 1.65)	62.25 (± 1.66)	53.84 (± 3.99)	43.21 (± 6.35)	17.44 (± 6.10)
Lactose	100	49.40 (± 9.95)	30.71 (± 6.98)	30.84 (± 6.42)	16.09 (± 5.71)	10.18 (± 5.04)
Adonitol	100	99.22 (± 1.45)	73.88 (± 8.09)	12.98 (± 8.02)	16.23 (± 5.04)	4.43 (± 5.91)
Water	100	88.42 (± 7.09)	50.16 (± 8.1)	21.47 (± 9.68)	0	0
Galactose	100	7.531 (± 16.84)	5.78 (± 12.92)	12.88 (± 8.9)	0	0
Tyrosine	100	94.58 (± 0.61)	31.74 (± 4.97)	0	0	0
Glycine	100	89.79 (± 0.96)	16.67 (± 9.49)	0	0	0
Proline	100	97.67 (± 2.61)	0	0	0	0
Methionine	100	88.16 (± 1.56)	0	0	0	0
Ornithine	100	85.60 (± 2.62)	0	0	0	0
Sodium deoxycholate	100	80.33 (± 5.04)	0	0	0	0
Cystein	100	0	0	0	0	0

### Desiccation of *P*. *putida* KT2440, its adherence to maize seeds after rehydration, and its capability to colonize the rhizosphere

**Fig 1:**
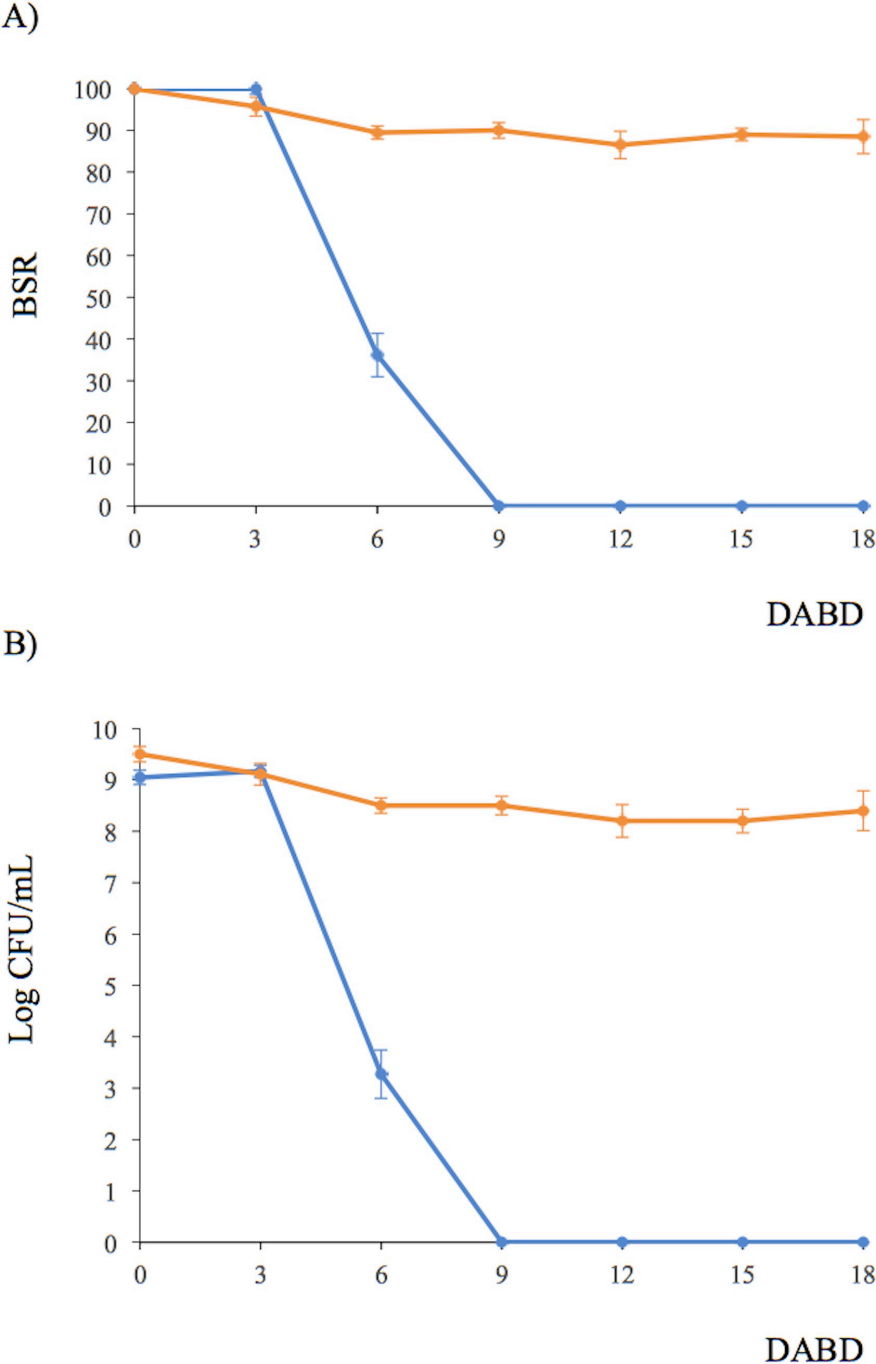
Bacterial survival ratio (A) and log CFU/mL (B) of *P*. *putida* KT2440 under desiccation stress (30°C and 50% RH). DABD means days after the beginning of desiccation.

**Fig 2:**
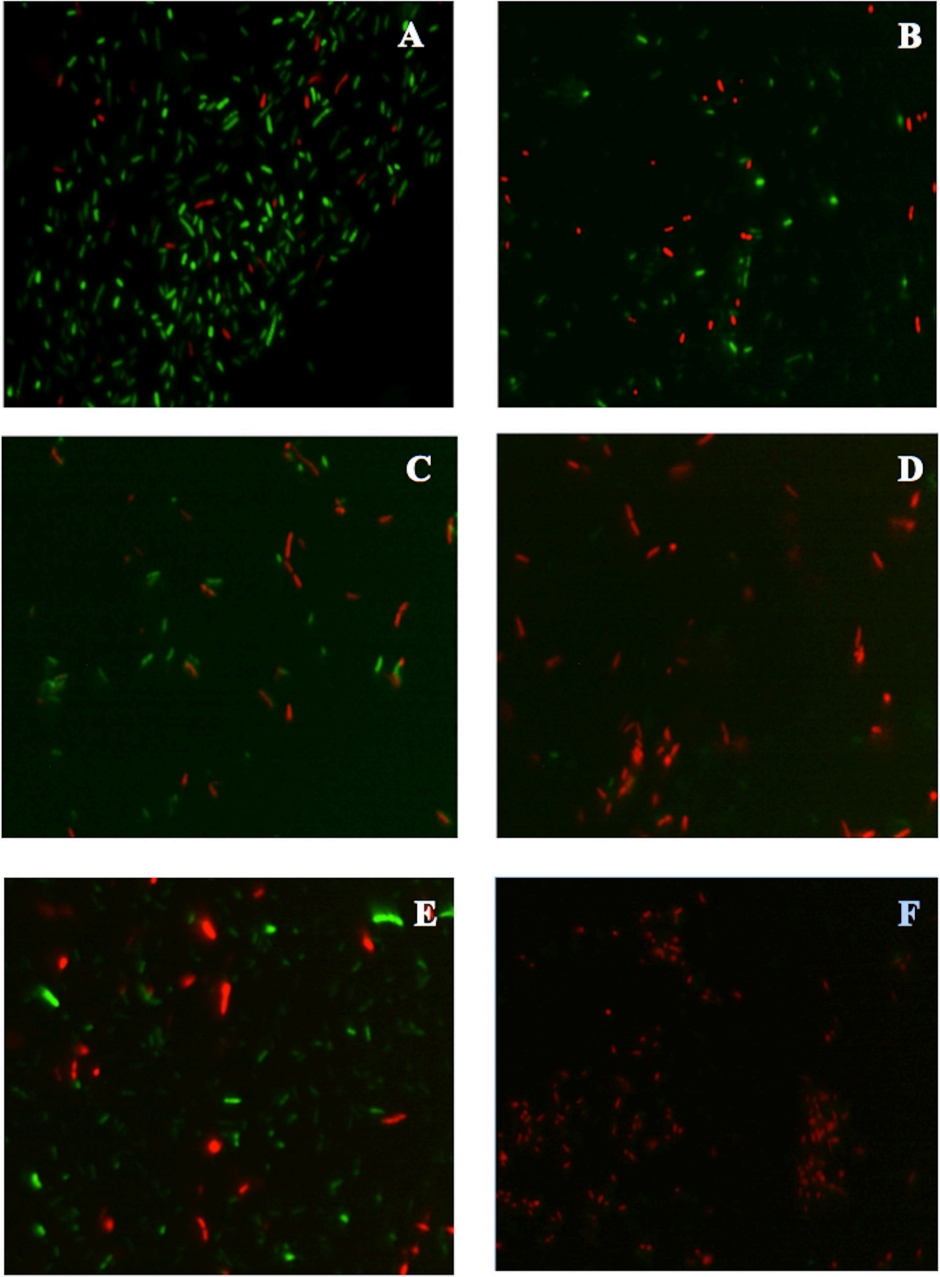
Fluorescence micrographs of *P*. *putida* KT2440 cells treated with the LIVE/DEAD BacLight Bacterial Viability Kit. (A) Bacterial cells before desiccation with trehalose (200 mM). (B) Bacterial cells before desiccation without a protector. (C) Bacterial cells protected with trehalose at 18 DABD. (D) Bacterial cells without a protector at 18 DABD. (E) Bacterial cells protected with trehalose adhered to germinated seeds after rehydration. (F) Bacterial cells without protection adhered to germinated seeds after rehydration. The samples were observed at 100×. Each image represents the MERGE of two captured images (green and red cells). The generation of MERGE images is shown in S1 and S3 Figs, and examples of MERGE cell analysis are shown in S2 and S4 Figs.

**Fig 3:**
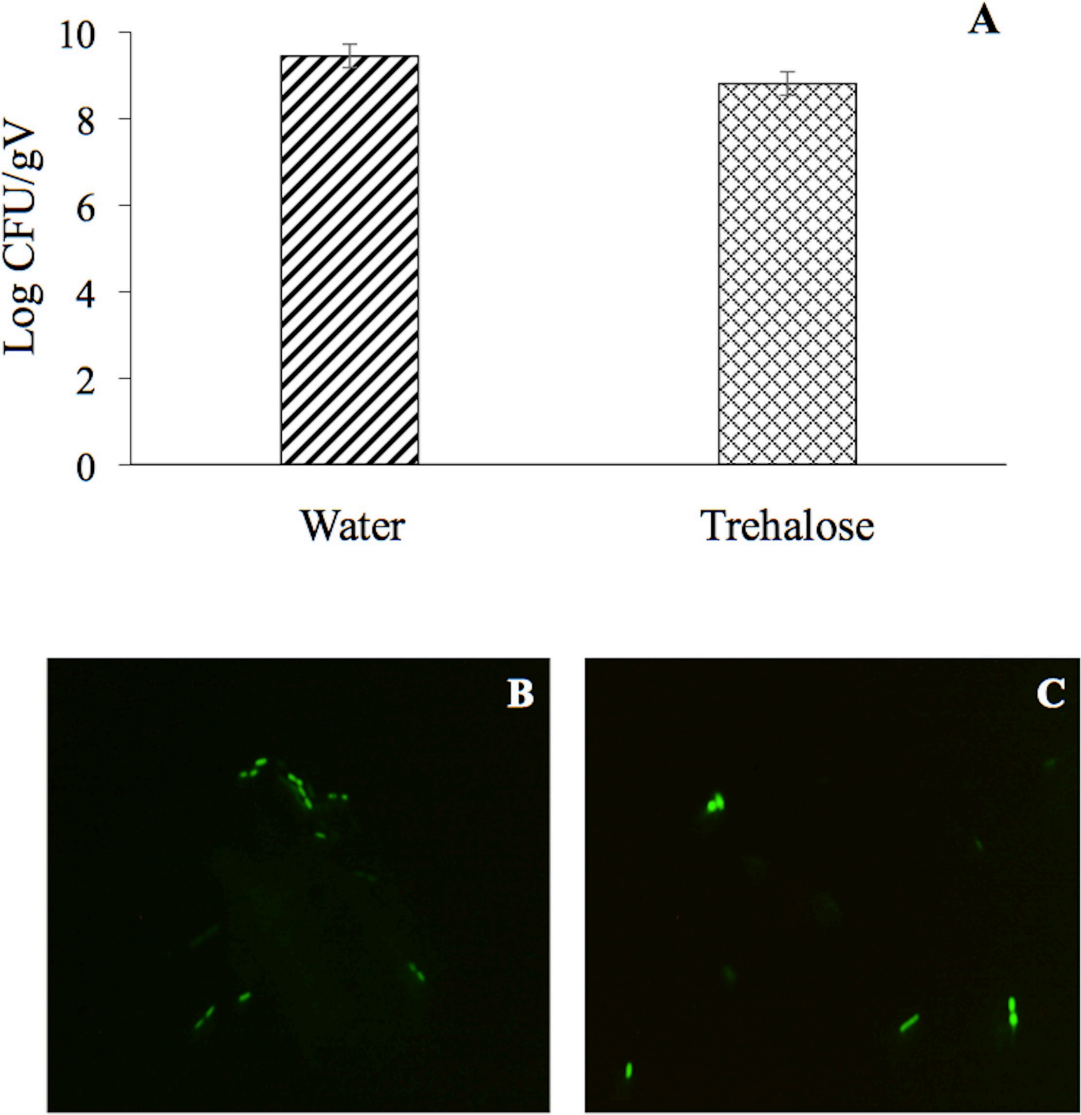
Rhizosphere colonization by *P*. *putida* KT2440. (A) Cell abundance from plants inoculated with rehydrated 18 DABD cells without protection in comparison to cell abundance from plants inoculated with rehydrated DABD cells in the presence of trehalose (200 mM). (B and C) Fluorescence micrographs of *P*. *putida* KT2440 using the LIVE/DEAD BacLight Bacterial Viability Kit. The samples were observed at 100×. (B) Cells obtained from the rhizosphere of plants inoculated with rehydrated 18 DABD cells without protection. (C) Bacterial cells obtained from the rhizosphere of plants inoculated with rehydrated 18 DABD cells in the presence of trehalose (200 mM). Each image represents the MERGE of two captured images (green and red cells). The generation of MERGE images is shown in S1D and S3D Figs, and examples of the analysis of cells in MERGE images are shown in S2D and S4D Figs.

**Fig 4:**
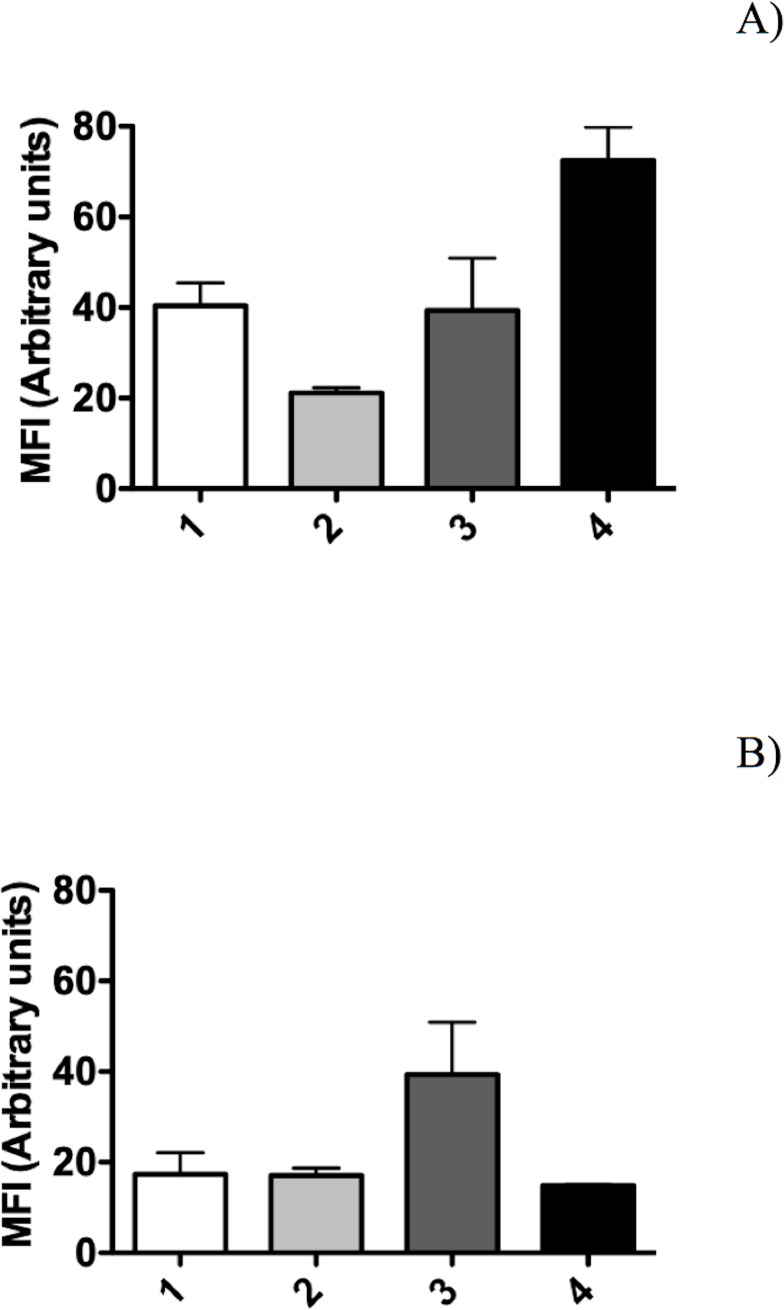
Mean fluorescence intensity (MFI) of *P*. *putida* KT2440 cells with protection (trehalose 200 mM). (A) Analysis of SYTO 9 and (B) analysis of propidium iodide. (1) Samples obtained from the bacterial suspension before desiccation, (2) cells at 18 DABD rehydrated for 20 min, (3) cells adhered to maize sprouts, and (4) cells from rhizosphere colonization.

**Fig 5:**
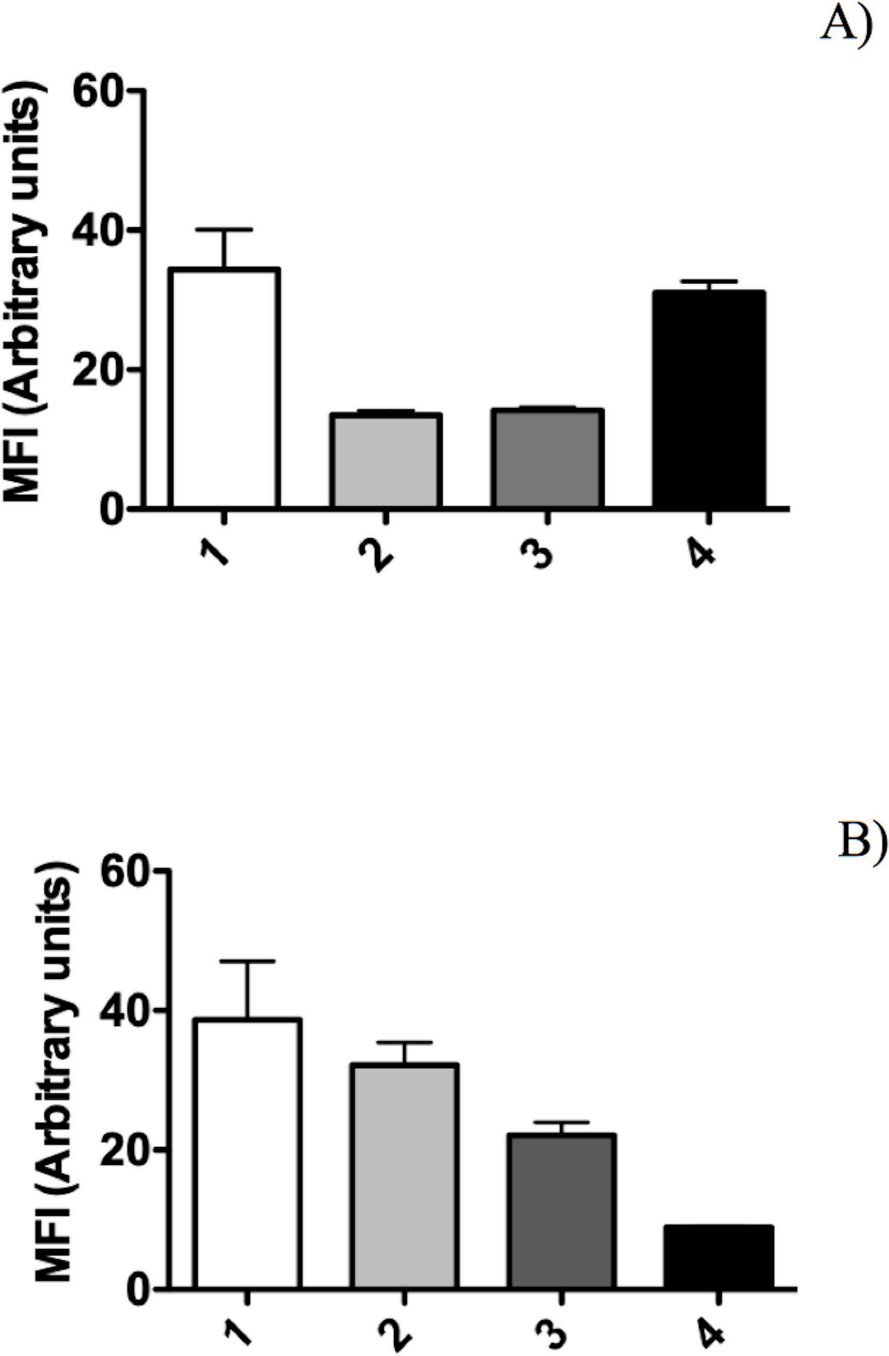
Mean fluorescence intensity (MFI) of *P*. *putida* KT2440 cells without protection. (A) Analysis of SYTO 9 and (B) analysis of propidium iodide. (1) Samples obtained from the bacterial suspension before desiccation, (2) cells at 18 DABD rehydrated for 20 min, (3) cells adhered to maize sprouts, and (4) cells from rhizosphere colonization.

The BSR of *P*. *putida* KT2440 decreased to an undetectable level after 9 DABD without a protector, but in the presence of trehalose as a protector, the bacterial abundance was approximately 10^8^ CFU/mL for all experimented time points (Fig 1). In this experiment, bacterial viability was evaluated in a suspension before desiccation and at 18 DABD using the LIVE/DEAD BacLight Bacterial Viability Kit. Before desiccation, the observed cells were green in both conditions, with and without trehalose (Fig 2A and 2B). However, at 18 DABD, the protected suspension contained green bacterial cells, and the nonprotected suspension contained only red bacterial cells (Fig 2C and 2D). Red cells lack an intact cell membrane and are conventionally scored as dead. At 18 DABD, the rehydrated samples were used to inoculate maize sprouts for 1 h. The number of bacteria in the suspensions did not change after interaction with sprouts (high numbers of bacteria in the presence of the protector and no detected bacteria in the absence of the protector). The sprouts were transferred to Falcon tubes containing 6.4 g of vermiculite, and 25 mL of water was added. The adherence of the bacteria was tested 2 h after sowing, and the bacterial abundance was 9.1×10^6^ CFU/sprouted seed in the treatment with trehalose, while bacteria were not detected in the treatment without a protector. Membrane integrity was tested with the LIVE/DEAD Kit, and several bacterial cells were observed to be green in the trehalose treatments (Fig 2E), while the nonprotected cells were all red (Fig 2F). Rhizosphere colonization by *P*. *putida* KT2440 was tested at 15 days post inoculation (dpi). Surprisingly, *P*. *putida* KT2440 colonized the rhizosphere of the plants in high numbers in both treatments: 4×10^8^ CFU/gV for the treatment with trehalose and 9×10^8^ CFU/gV for the treatment without a protector (Fig 3A). The recuperation of bacterial cells in the rhizosphere of plants derived from seeds on which bacteria were not detected after desiccation could mean that the bacteria entered a viable but nonculturable state during the desiccation process (treatment without the use of protectors) and had returned to a culturable state when the bacteria interacted with the plants. The membrane integrity of the bacteria colonizing the plant rhizosphere was evaluated, and all observed bacteria were green in both treatments, suggesting that the membranes of the cells, from the treatment without protection were recovered in terms of their integrity and that the crossing of propidium iodide was prevented during their interaction with the plants (Fig 3B and 3C). The MFI analysis corroborated the observations of membrane integrity restoration after bacterial colonization because the MFI of SYTO 9 increased with respect to that of propidium iodide both in the presence and absence of the protector (Figs 4 and 5).

### Desiccated nonculturable *P*. *putida* KT2440 returns to a culturable state after rehydration with plant exudates or under prolonged rehydration

**Fig 6:**
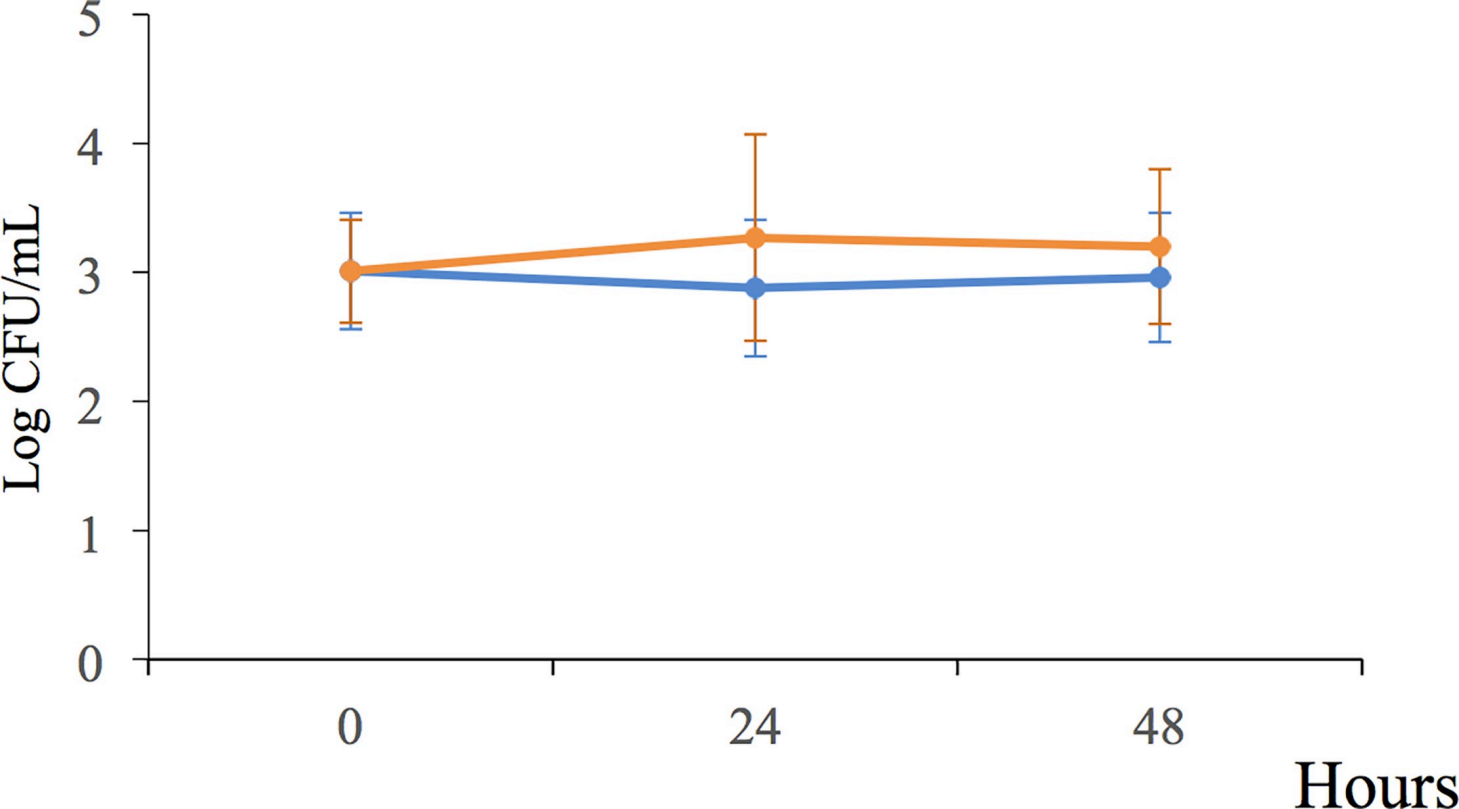
Bacterial behavior of *P*. *putida* KT2440 cells rehydrated with water (orange line) or in the presence of root exudates (blue line) under static conditions for 48 h.

To explore whether the exudates of plants return *P*. *putida* KT2440 to a culturable state, cells in the stationary phase were desiccated until 18 DABD and rehydrated three different suspensions: 1) maize sprout exudates, 2) maize root exudates following 12 days of growth, and 3) water only as a control. Rehydration was carried out for 20 min and 1, 3, 6, 9, 12, 24, 27, 30 and 48 h, and the bacterial abundance was determined. As expected, the bacterial abundance had decreased to nondetectable levels at 18 DABD, but the bacteria returned to a culturable state after rehydration with plant root exudates or with only water; interestingly, this return was faster in the presence of exudates; in contrast, in water, the bacterial abundance was similar to that of the samples rehydrated with the exudates for up to 48 h (Table 3). It is noteworthy that the plant root exudates obtained from the early stages were able to accelerate the return to a culturable state of the *P*. *putida* KT2440 cells (Table 3). During rehydration, the bacterial suspension in the microtubes remained static, and independent experiments showed that the bacterial cells were unable to duplicate under this condition in the presence of both exudates and only water (Fig 6). Therefore, the increase in *P*. *putida* KT2440 observed after the rehydration of desiccated cells corresponds to cells returning to a culturable state but not to active growth under static rehydration. Because the bacteria returning to a culturable state reached levels of cultivability similar to that observed for the bacteria rehydrated with the plant root exudates, later experiments were conducted under prolonged water rehydration.

**Table 3:** Number of cells of *P*. *putida* KT2440 (log CFU/mL) 18 DABD and rehydrated with root exudates of maize.

Maize root exudates	Time of rehydration								
	20 min	3 h	6 h	9 h	12 h	24 h	27 h	30 h	48 h
Maize sprout	0	0	0	0	3.09 ±0.15	4.81 ±0.19	6.09 ±0.14	6.21 ±0.10	7.11 ±0.19
12 days of growth of plant	0	0	0	0	3.21 ±0.41	4.78 ±0.26	6.14 ±0.29	6.62 ±0.09	7.32 ±0.23
Water control	0	0	0	0	0	4.34 ±0.16	5.46 ±0.29	5.34 ±0.16	7.39 ±0.36

### Evaluating the membrane integrity of *P*. *putida* KT2440 after desiccation and rehydration

**Fig 7:**
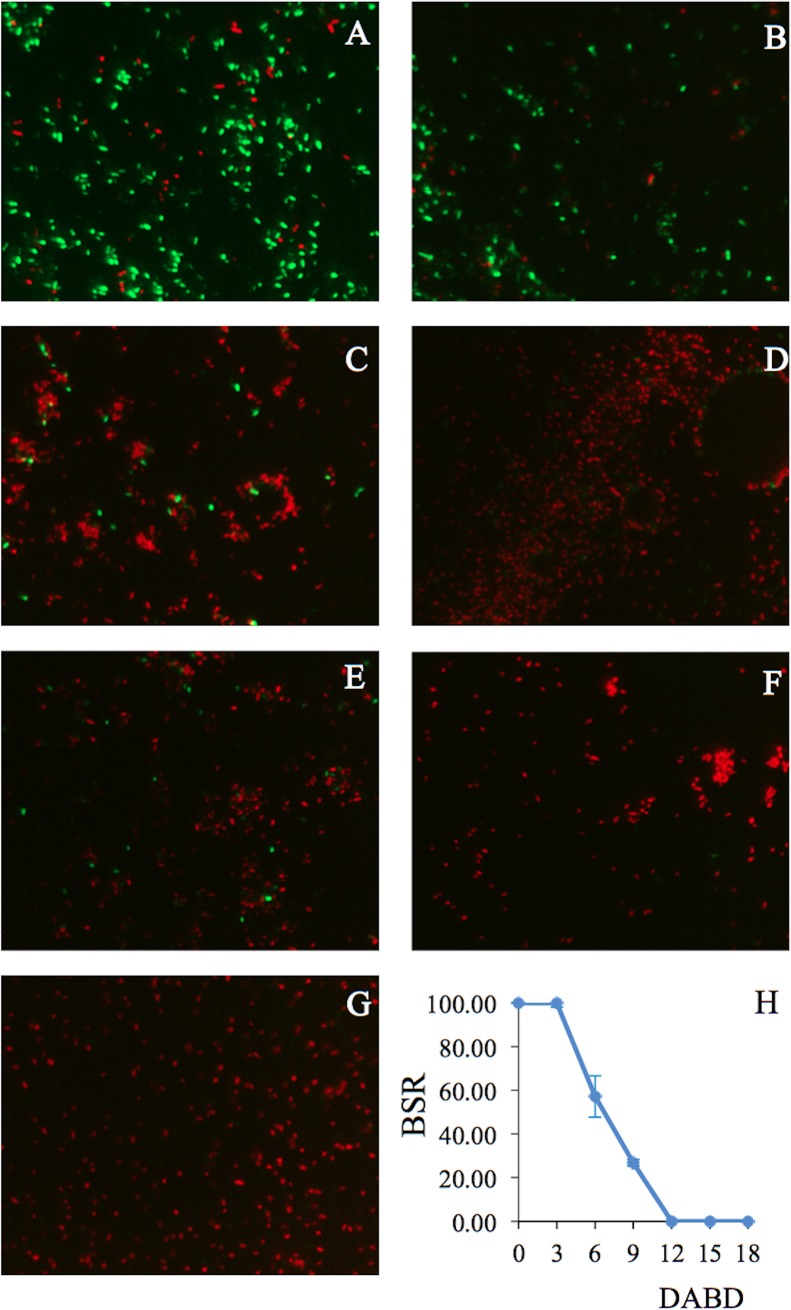
Fluorescence of *P*. *putida* KT2440 cells desiccated for 18 days and stained with the LIVE/DEAD BacLight Bacterial Viability Kit. (A) Cells before desiccation and (B) 3, (C) 6, (D) 9, (E) 12, (F) 15, and (G) 18 DABD. Each image represents the MERGE of two captured images (green and red cells). The samples were observed at 100×. The generation of MERGE images is shown in S5 Fig, and examples of the analysis of MERGE images are shown in S6 Fig. (H) The BSR of *P*. *putida* KT2440 during desiccation.

**Fig 8:**
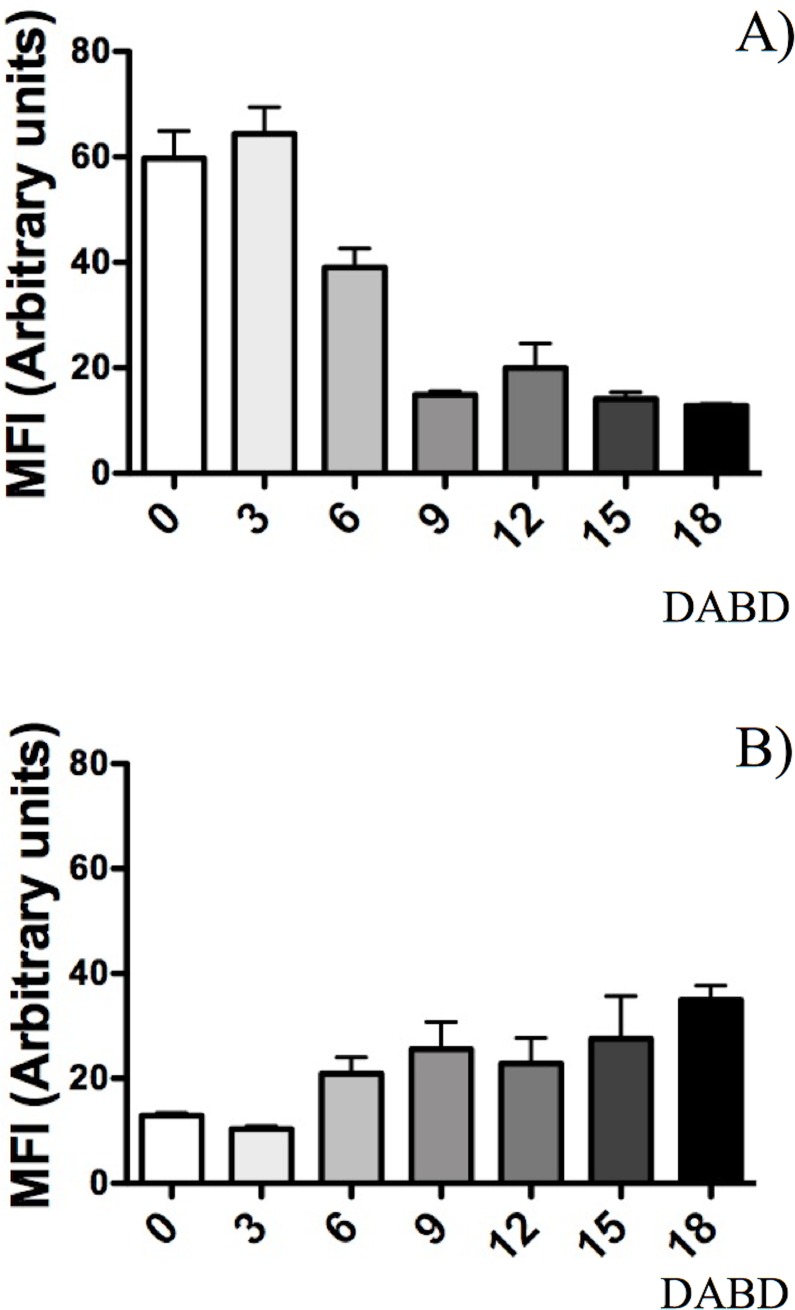
Mean fluorescence intensity (MFI) of the same images as in Fig 7. (A) Analysis of SYTO 9. (B) Analysis of propidium iodide.

**Fig 9:**
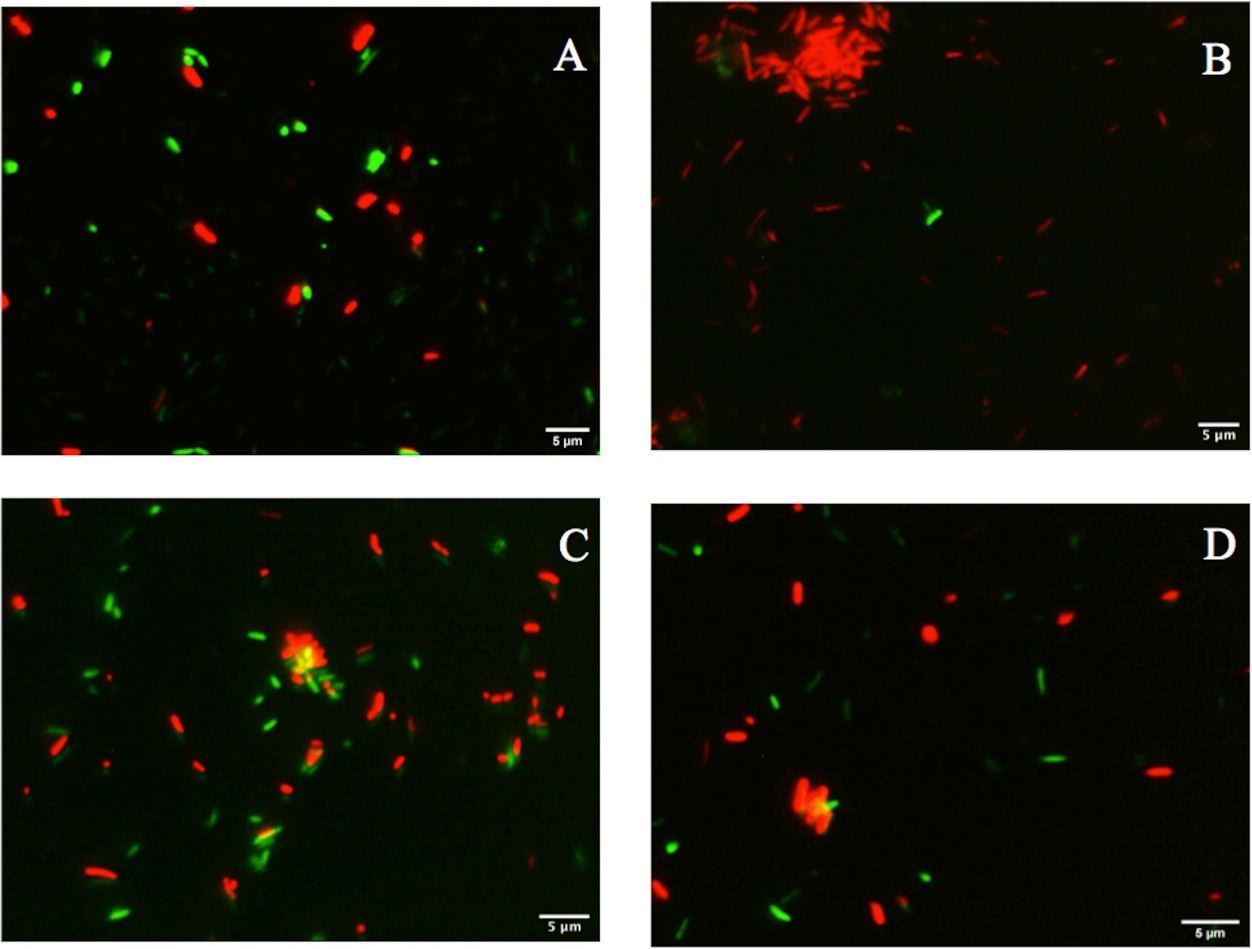
Fluorescence of *P*. *putida* KT2440 cells desiccated for 18 days and stained with the LIVE/DEAD BacLight Bacterial Viability Kit. (A) Cells before desiccation, (B) cells at 18 DABD rehydrated for 20 min, (C) cells at 18 DABD rehydrated for 24 h, (D) cells at 18 DABD rehydrated for 48 h. The samples were observed at 100×. Each image represents the MERGE of two captured images (green and red cells). Generation of MERGE images is shown in S7 Fig, and examples of analysis of MERGE images are shown in S8 Fig.

**Fig 10:**
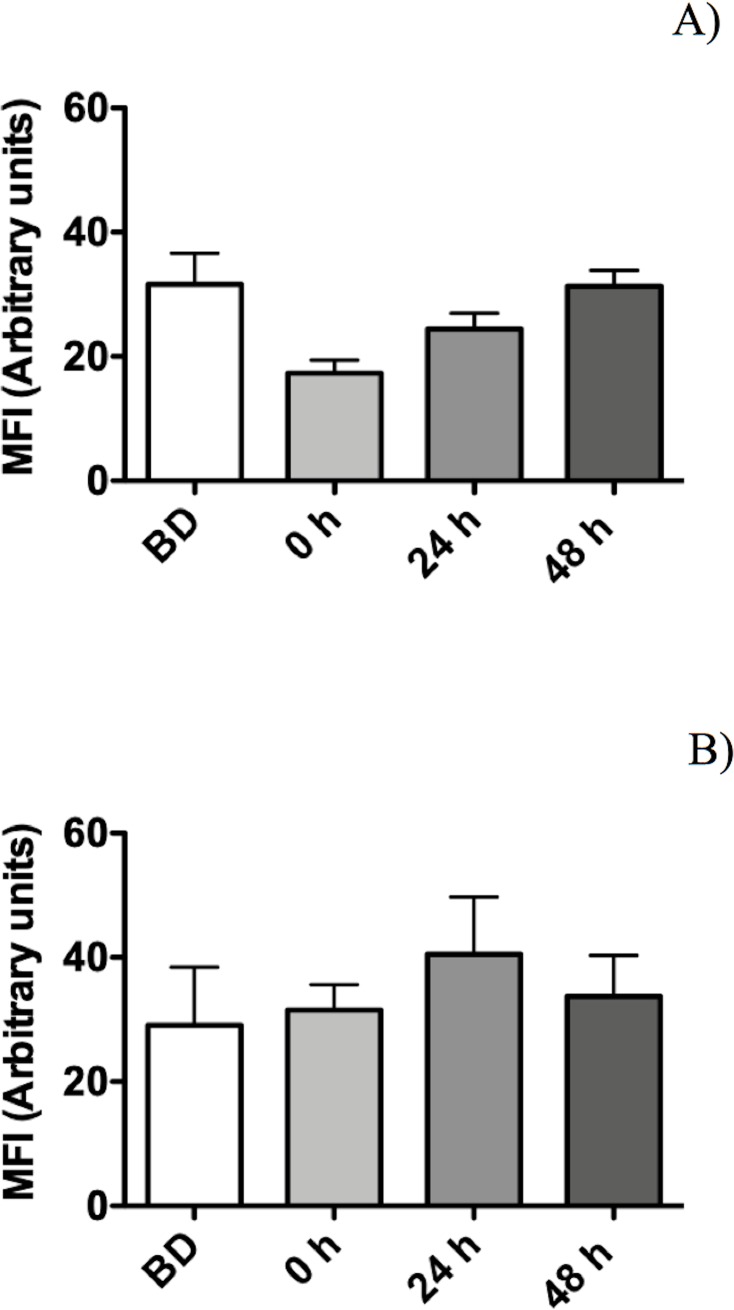
Mean fluorescence intensity (MFI) of the same images as in Fig 9. (A) Analysis of SYTO 9. (B) Analysis of propidium iodide.

**Fig 11:**
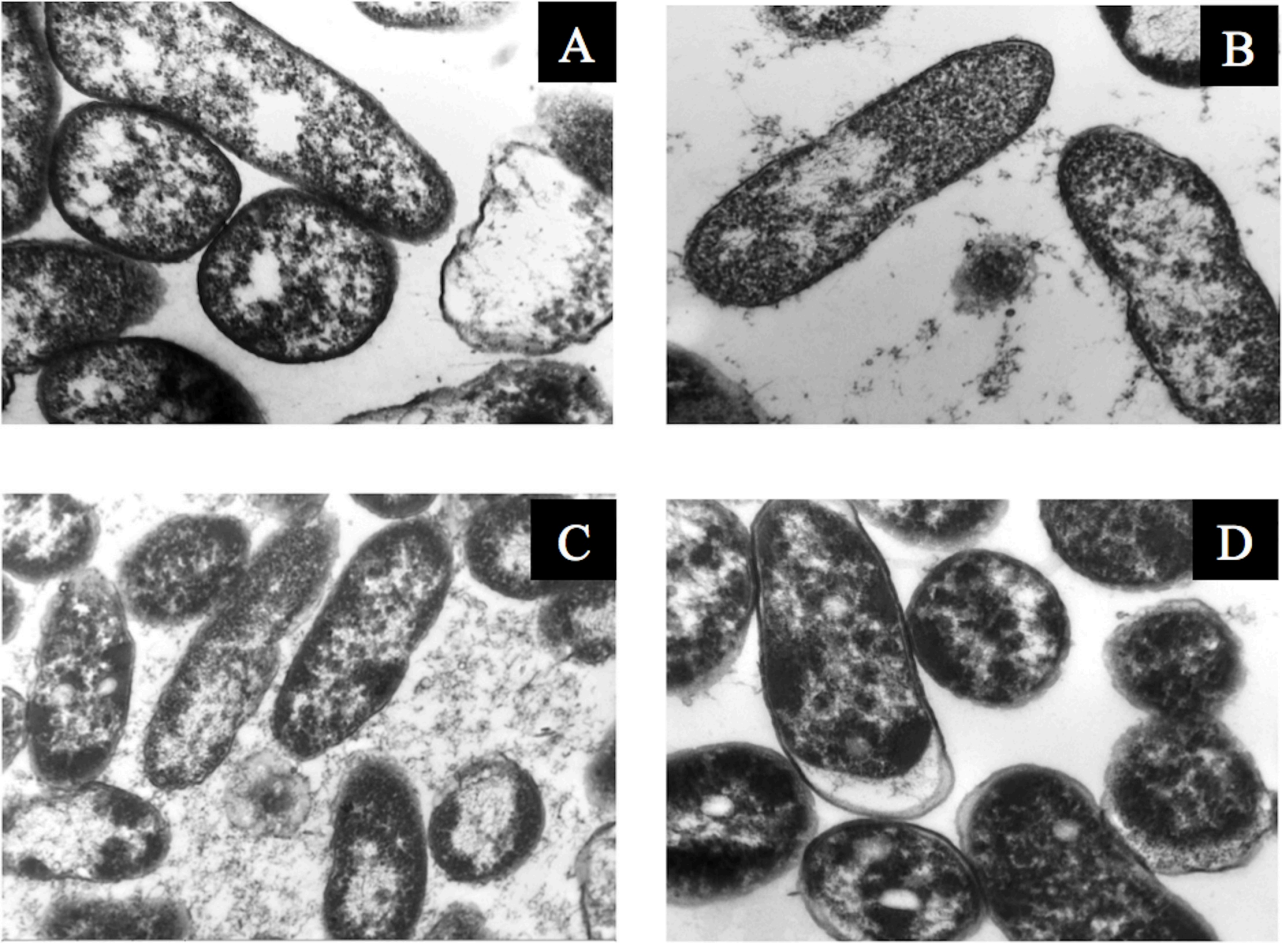
Transmission electron microscopy of *P*. *putida* KT2440. (A) Before desiccation, 50,000×, (B) at 6 DABD, 30,000×, (C) at 12 DABD, 30,000× and (D) at 18 DABD, 50,000×.

Bacterial membrane damage caused during desiccation and rehydration was related to bacterial viability and cultivability. *P*. *putida* KT2440 was desiccated at 30°C and 50% RH until 18 DABD, and every 3 days, the number of culturable bacteria and membrane integrity were evaluated. Before desiccation, the bacterial abundance was approximately 4.5×10^8^ CFU/mL, with a BSR of 100 (Fig 7H), and the majority of the observed cells were green when the LIVE/DEAD BacLight Bacterial Viability Kit was used, indicating the presence of healthy membranes (Fig 7A, S5A and S6A Figs). The BSR decreased to 0 at 12 DABD (Fig 7H), and the number of green bacteria decreased with a concomitant increase in red bacteria (Fig 7), in accordance with the MFI analysis (Fig 8). This result indicates that the bacterial membrane is damaged during desiccation stress, which is associated with the inability of the bacteria to grow in the culture media after a short rehydration period. At 18 DABD, the samples were rehydrated for 24 and 48 h, and an increase in the number of culturable bacterial cells to 2.3×10^4^ and 3.2×10^7^ CFU/mL, respectively, was observed, with a similar increase in the number of green bacterial cells, which indicated that membrane reparation had occurred (Fig 9, S7 and S8 Figs). The increase in green intensity was supported by the MFI values (Fig 10A); interestingly, the MFI values associated with propidium iodide were similar to those found under rehydration (Fig 10B). Bacterial staining using toluidine blue showed a decrease in bacterial size among the desiccated bacteria (S9 Fig). TEM observation of the bacterial cells during desiccation showed that the bacteria suffered a retraction of the cell cytoplasm with a concomitant incremease of periplasmic space (Fig 11).

### Desiccation of *P*. *putida* KT2440::*gfp* (green fluorescent protein)

**Fig 12:**
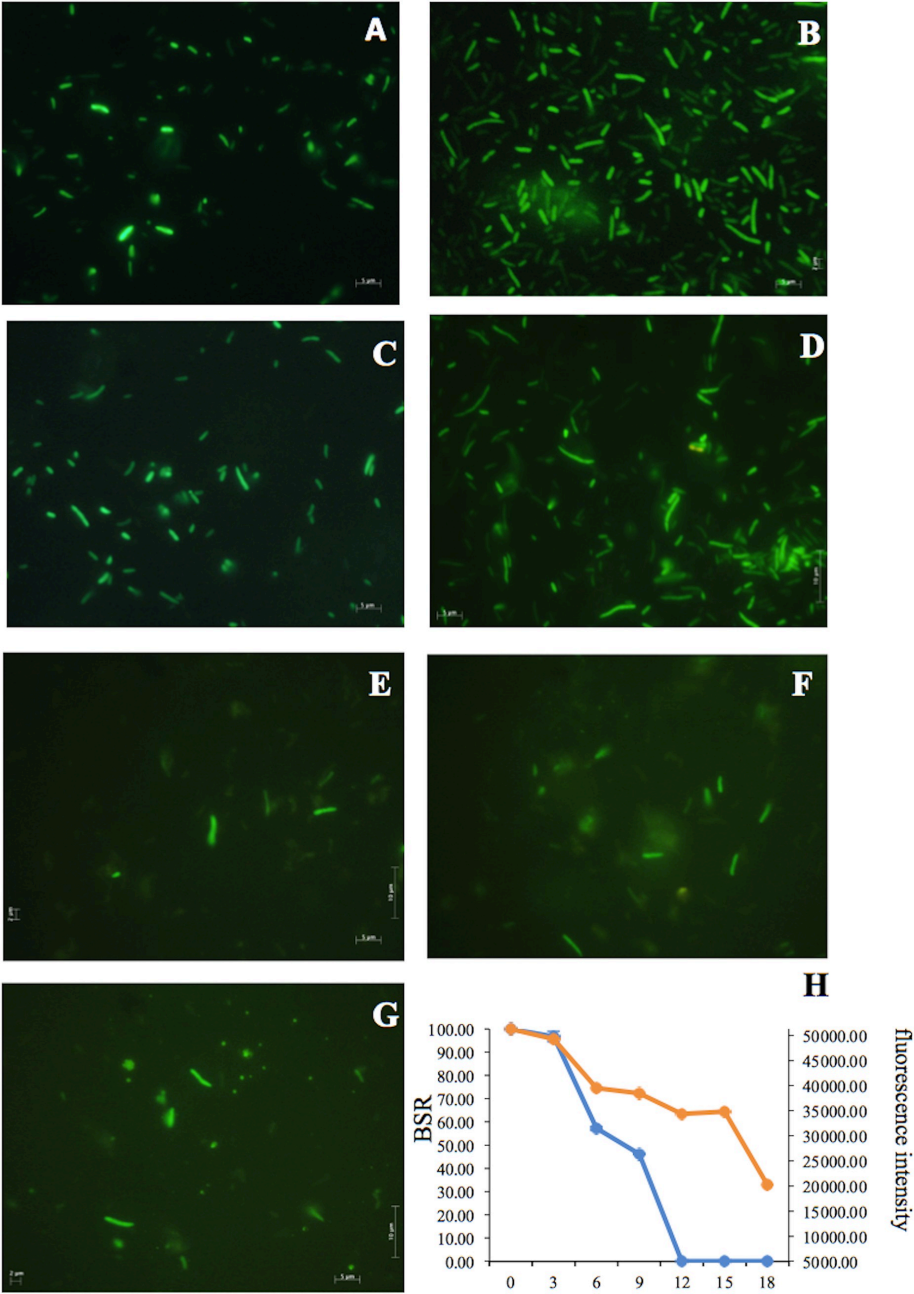
Fluorescence of *P*. *putida* KT2440 tagged with GFP desiccated for 18 days. (A) Cells before desiccation and (B) 3, (C) 6, (D) 9, (E) 12, (F) 15, and (G) 18 DABD. The samples were observed at 100×. (H) The BSR of *P*. *putida* tagged with GFP during desiccation (blue line) and fluorescence intensity of *P*. *putida* tagged with GFP during desiccation (orange line).

A miniTn7-GFP mutant strain of *P*. *putida* KT2440, which constitutively expresses the green fluorescent protein [39], was used to explore whether this protein is expressed by bacterial cells after desiccation. The desiccation tolerance of *P*. *putida* KT2440::*gfp* was similar to that of the wild-type strain; this strain was nonculturable at 12 DABD (Fig 12H), but under fluorescence microscopy, cells of *P*. *putida* tagged with GFP were green during different stages of desiccation (Fig 12), indicating constitutive expression and active synthesis of this protein. However, the fluorescence intensity of the desiccated bacterial cells partially decreased until 18 DABD (Fig 12H).

### Bacterial gene expression of some constitutive genes before and after desiccation and rehydration

RNA was extracted from nondesiccated and desiccated-rehydrated cells to evaluate the active expression of genes from culturable and nonculturable cells. Desiccated cells from 18 DABD and, in some cases, 40 DABD were rehydrated for 20 min or 24 h. The explored genes were 16S rRNA, *rpoN* (housekeeping), *mutL*, *mutS* (codifying proteins from the mismatch repair complex), and a gene codifying an outer membrane protein widely distributed in *P*. *putida* KT2440 (*oprH*). All evaluated genes were expressed by cells both before desiccation and after desiccation-rehydration independent of the time of desiccation or rehydration (S10 and S11 Figs), which means that the desiccated-rehydrated cells were alive at all evaluation times, even though they had membrane damage or were nonculturable.

**Fig 13:**
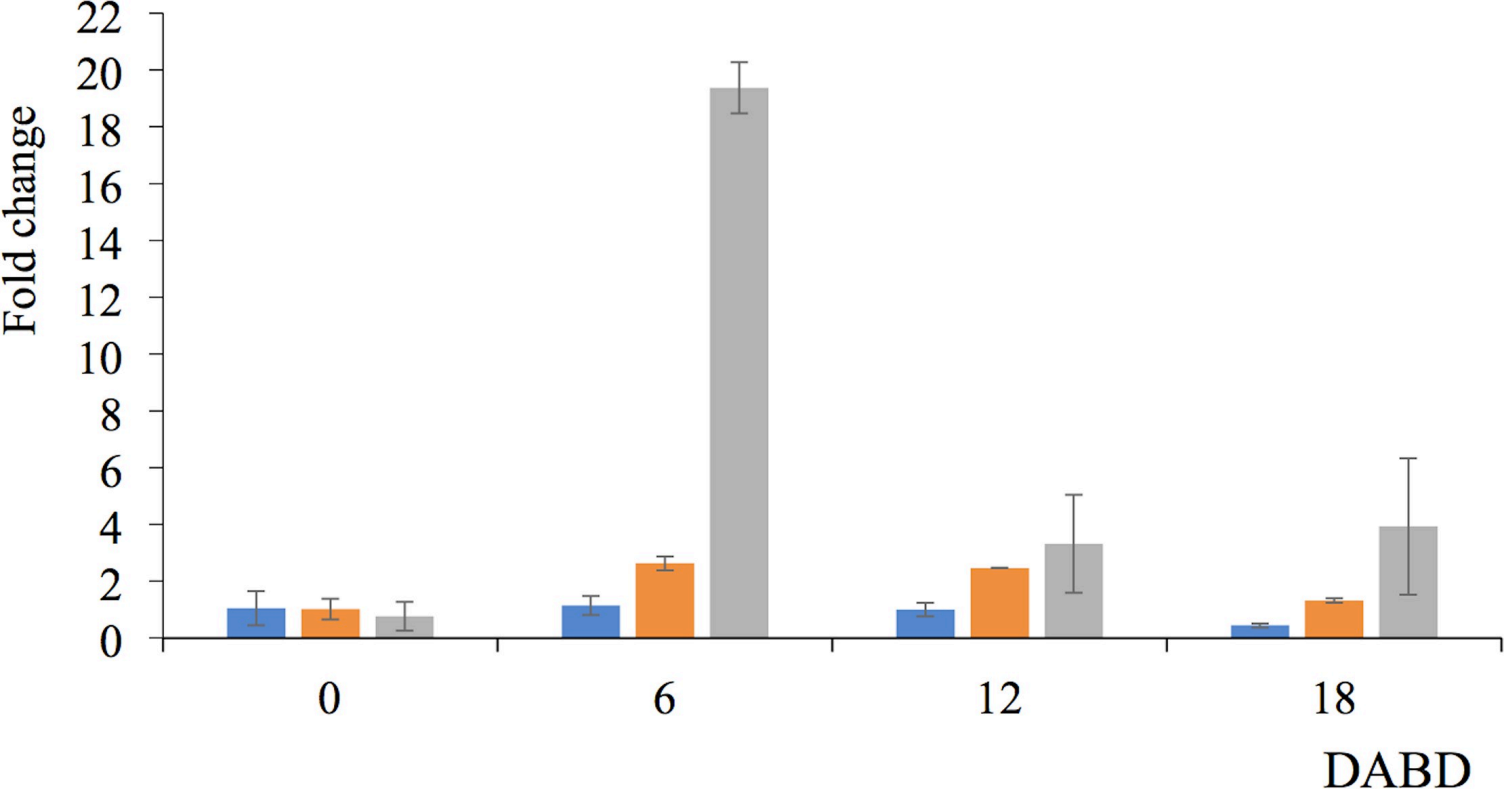
**Gene expression levels of *mutL* (blue bar), *mutS* (orange bar) and *oprH* (gray bar) obtained by RT-qPCR**. Before desiccation (0) and 6, 12 and 18 DABD.

The level of expression of the *mutL*, *mutS* y *oprH* genes from *P*. *putida* KT2440 was quantified before desiccation and at 6, 12 and 18 DABD with the RT-qPCR method. Interestingly, *oprH* gene expression was markedly increased at 6 DABD (19.37 times), and the expression declined at 12 or 18 DABD (Fig 13), but it was higher than the levels observed before desiccation. The level of expression of the *mutS* gene increased more than 2 times at 6 and 12 DABD and returned to levels similar to those of nondesiccated cells at 18 DABD. The level of expression of the *mutL* gene was constant, but it decreased at 18 DABD.

## Discussion

*P*. *putida* KT2440 has high agro-biotechnological potential [4,6,42]. According to our results, this bacterium is very sensitive to air desiccation, and the benefits of a sensitive bacterial species could be lost after desiccation occurs in the environment [20,21]. Bacterial tolerance to desiccation is a key factor in designing stable inoculants because tolerant bacteria can adhere to seeds, tolerate desiccation in field soils, and colonize plant roots after rehydration when environmental conditions are favorable, maintaining their ability to improve plant growth [6,20]. Therefore, the high tolerance of *P*. *putida* KT2440 and studies about how this bacterium tolerates desiccation are very important to exploit its capabilities in fields. Disaccharides have been reported to be good protectors of *P*. *putida* KT2440 under freeze-drying conditions [12,13]. However, in our work, only nonreducing disaccharides were able to effectively protect *P*. *putida* KT2440 cells from the effects of air desiccation. The best protector was trehalose, resulting in high cultivability in this bacterium, and this disaccharide could be used to formulate bacterial powder inoculants. Although trehalose produced by genetically modified cells of *P*. *putida* KT2440 was unable to protect the cells from the effects of freeze-drying [13], this disaccharide was shown to be able to protect *P*. *putida* KT2440 from the effects of the freeze-drying process when this sugar was added at 200 mM [12], as was also observed in terms of protection against air desiccation in our work.

Trehalose facilitated high bacterial culturability even at 18 DABD; however, in the treatments without protection, the number of culturable cells decreased to a nondetectable value after 12 DABD. Therefore, we hypothesize that only rehydrated protected cells can adhere to roots and colonize the rhizosphere of maize plants. As expected, only protected cells were detected in the adhesion assays. Surprisingly, the number of bacteria colonizing the rhizosphere of maize plants was similarly high in the protected and nonprotected cell treatments. We propose that during desiccation, *P*. *putida* KT2440 enters a nonculturable state as a strategy to cope with stress. However, the bacterial cells return to a culturable state after interacting with plant roots, likely because the exudates of plants activate mechanisms related to this return. To determine whether root exudates allow *P*. *putida* KT2440 to return to cultivability, the bacterial cells were desiccated until 18 DABD, and they were rehydrated with only water or with maize root exudates; *P*. *putida* KT2440 cells returned to a culturable state after rehydration with plant root exudates faster than they did with only water, suggesting that some compounds or molecules from exudates favor the return to a culturable state. Studies have shown that when *Bacillus atrophaeus* UCMB-5137 grows in the presence of corn exudates, the expression of genes related to stress and detoxification is stimulated [43]; it is probable that the maize root exudates favor the expression of genes necessary for the return to a culturable state for *P*. *putida* KT2440 after rehydration. Among the compounds present in the exudates of corn plants are nitrogen compounds, fatty acids, organic acids, sugars, volatile compounds, steroids, terpenoids and other substances [44,45]. It will be interesting to carry out future trials to clarify which compounds favor the quickest return to a culturable state in *P*. *putida* KT2440 following the rehydration of desiccated cells. The return to a culturable state occurs when bacteria find favorable conditions and when the stress is completely withdrawn [25], and this could explain why in our work bacterial cells were also able to return to a culturable state after prolonged rehydration.

Bacteria in the VBNC state can be identified using techniques that determine whether they are metabolically active even if they are not culturable. These methodologies include assays with the LIVE/DEAD BacLight Bacterial Viability Kit, which identifies damage to the plasmatic membrane [25], studies of the green fluorescent protein (GFP), which is constitutively expressed by nonculturable bacteria [30,46], and gene expression studies using molecular tools, such as RT-PCR and RT-qPCR [23,47]. In the present work, we used these techniques to evaluate whether *P*. *putida* KT2440 remain active in the VBNC state during desiccation. By using the LIVE/DEAD BacLight Bacterial Viability Kit, it was observed that there was an increase in the number of red bacteria and that the propidium iodide MFI values were greater than the MFI values of SYTO 9, indicating that the injury to membranes of *P*. *putida* KT2440 increases as the duration of desiccation progresses. During desiccation, bacterial membrane changes in the composition of fatty acids and experience alterations in its fluidity [48], and destabilization could allow propidium iodide to cross the membrane and the red staining of cells. According to the kit and other works, red cells are considered dead [49]. However, our results showed that the red bacterial cells from 18 DABD (after rapid rehydration) were nonculturable, but they returned to a culturable state after prolonged rehydration or rehydration in the presence of exudates, indicating that the red cells never died.

Interestingly, under prolonged rehydration (24 and 48 h), the bacteria decreased in terms of the intensity of their red coloration, as indicated by the microscopic analysis and MFI values, and increased in terms of the intensity of their green coloration. This result indicates that the penetration of the bacterial cells by propidium iodide decreased, likely due to the recovery of membrane integrity, and again, that the crossing of propidium iodide was prevented. In fact, in association with plants, the red intensity declined, and only green cells were observed, indicating the total recovery of the integrity of the membranes. However, the red staining by propidium iodide do not necessarily mean “dead cells”. It was reported that viable *P*. *putida* (ATCC 12633) cells were stained by propidium iodide suggesting that the use of LIVE/DEAD BacLight Bacterial Viability kit may give a confusing result in determining live cells [50]. We propose that additional studies like TEM, changes in membrane fluidity, changes in membrane potential and changes in membrane phospholipid composition will be required to clarify the level of membrane damage in our viable but non-culturable bacterial cells subjected to desiccation-rehydration process.

The first assay to verify the metabolic activity of nonculturable cells involved the desiccation of *P*. *putida* KT2440::*gfp*, a strain that constitutively expresses the green fluorescent protein (GFP) [39]. *P*. *putida* KT2440::*gfp* tolerated air desiccation at a level similar to that of wild-type and the presence of nonculturable cells were observed after 12 DABD. However, in all the experiments, fluorescent cells were observed without an apparent decrease in intensity at 15 DABD, with a slight decrease at 18 DABD. This result suggests the occurrence of active metabolic activity in rehydrated nonculturable cells. GFP studies have also been carried out with other models to evaluate the VBNC state of cells [46,51]. In our work, the fluorescence intensity and microscopic observation of green cells confirmed the viability and cellular activity of nonculturable rehydrated *P*. *putida* KT2440::*gfp* cells.

Molecular tools such as RT-PCR and RT-qPCR have been widely used to determine cellular viability because the average lifetime of mRNA is approximately 3 to 5 min. Thus, amplified RNA molecules arise from active and recent transcription and are an excellent indicator of metabolic activity in VBNC cells [52,53]. To increase our knowledge of the cellular activity of nonculturable rehydrated *P*. *putida* KT2440 cells, we monitored the expression of housekeeping genes (*RpoN* and 16S rRNA), genes encoding proteins from the mismatch repair complex (*mutL* and *mutS*), and a gene encoding an outer membrane protein (*oprH*) both before desiccation and at 18 DABD. All explored genes in the present work were amplified from RNA samples after a short period of rehydration of nonculturable *P*. *putida* KT2440 cells, indicating active expression of the genes in that state and consequently active metabolic activity. 16S rRNA has been widely used to explore the VBNC state of bacteria [54,55], and in our work, it was the first gene selected to evaluate cell activity, as ribosomal genes were observed to be active after rehydration. The gene *rpoN* encodes the σ^54^ factor from RNA polymerase and regulates the expression of several genes [56–58]. This housekeeping gene (*rpoN*) was used as a reference gene for the RT-qPCR in this study. After amplification with RT-qPCR, it was observed that the *oprH*, *mutL* and *mutS* genes in nonculturable rehydrated *P*. *putida* KT2440 cells were expresed. The *oprH* gene encodes one of the most abundant membrane proteins of the genus *Pseudomonas*, which functions mainly as an aquaporin and a drug transporter efflux protein and is important for the maintenance and support of the membrane [59]. Expression of the *oprH* gene has been observed to be highly induced by high oxygen pressure conditions, likely as a consequence of plasmatic membrane destabilization caused by this type of stress [59]. In our work, the expression of the *oprH* gene highly increased at 6 DABD, suggesting that under desiccation conditions, the destabilization of cell membranes occurs, which is supported by the increase in red bacterial cells observed under fluorescence microscopy. Although *oprH* gene expression decreased at 12 and 18 DABD, this expression was higher than that observed for the *mutL* and *mutS* genes; therefore, we propose this gene as an indicator of viability in *P*. *putida* KT2440 under desiccation stress.

The *mutL* and *mutS* genes codify proteins from the mismatch repair complex, which acts in DNA replication and is also involved in the signaling process to prevent chemical damage that could occur under air desiccation, such as oxidation, Maillard reactions, and DNA damage [18,60]. Therefore, *mutL* and *mutS* could be fundamental genes involved in DNA reparation, and the study of the rehydration of desiccated *P*. *putida* KT2440 cells was carried out. Our results show a decrease in the expression of the *mutL* gene in nonculturable cells after rehydration, but this gene was expressed over time. For the *mutS* gene, the expression was similar in culturable and nonculturable cells. Therefore, these genes could be involved in DNA reparation in cells damaged by desiccation. In our work, RT-PCR of the *gfp* gene from nonculturable cells was not performed, and it will be interesting to carry out such assays in the future.

## Conclusions

*Pseudomonas putida* KT2440 has very low tolerance to air desiccation in comparison to other bacteria; however, trehalose protects it from stress and could be used to design powder-stable inoculants with the capability to maintain the viability of the strain without the use of a vacuum, decreasing the cost of production. Furthermore, this bacterium enters the VBNC state during desiccation without protector addition, which could be a strategy to mitigate the stress. *P*. *putida* KT2440 cells in a desiccated state have a decreased cell size and retracted cytoplasm. Desiccation also causes damage to the membranes of *P*. *putida* KT2440, and these membranes can apparently be repaired after prolonged rehydration or rehydration in the presence of plant root exudates. After a short rehydration period, the bacterial cells are not capable of growth in culture media (VBNC state); however, the transcription of several genes remains active under these conditions in the wild-type strain, and GFP activity was also detected in cells targeted with the *gfp* gene, showing active metabolic activity after the rehydration of desiccated cells. Data from the present work support that during desiccation, this bacterial strain suffers damage to its membranes and enters the VBNC state, and the bacterial cells return to a culturable state after their interaction with plant roots or prolonged rehydration when the membranes are repaired. Therefore, this work will contribute to the development of bacterial inoculants containing live bacteria in a VBNC state that could return to a culturable state after interaction with root plants when water conditions are completely favorable to germination, leading to successful colonization and the maintenance of their beneficial effects.

This work opens new avenues of research in the field of bacterial survival under desiccation stress and breaks the paradigm of the bacterial membrane integrity concept. Today, people think that if the membrane is affected, then the bacteria are dead, but we showed that even though the *P*. *putida* KT2440 membrane was affected, this bacterium was able to return to a culturable state if environmental conditions were favorable for its growth. The results of the present work could be taken into account to design bacterial inoculants for application in agriculture, by using live bacteria in a VBNC state that can return to a culturable state after the interacting with plant roots when the conditions are favorable for germination and colonization. The inoculant industry adheres to some norms regarding the number of bacteria in inoculant formulations that indicate that when the number decreases below a specified threshold, the inoculant should be discarded. However, whether the bacteria are truly dead or remain only nonculturable should be considered because the bacteria could return to a culturable state after their interaction with plants.

## Supporting information

S1 Fig Fluorescence microscopy of *P. putida* KT2440 cells treated with the kit “Live/Dead BacLight Bacterial Viability” from treatments desiccated in presence of trehalose (200 mM). A) Bacterial cells before desiccation. B) Bacterial cells at 18 DABD. C) Bacterial cell adhered to germinated seeds. D) Rhizosphere colonization of *P*. *putida* KT2440 from plants inoculated with rehydrated cells of 18 DABD. Each row shows two captured imagens; the images of column SYTO 9 were taken at filter with excitation 420–490 nm, images of column propidium iodide were taken at filter excitation 500-550nm. MERGE correspond to combination of both images (SYTO 9 and propidium iodide).
(PDF)

S2 Fig MERGE imagens and histograms that represent distribution of SYTO 9 and propidium iodide from cells random selected. Treatments desiccated with trehalose. A) Bacterial cells before desiccation. B) Bacterial cells at 18 DABD. C) Bacterial cell adhered to germinated seeds. D) Rhizosphere colonization of *P*. *putida* KT2440 from plants inoculated with rehydrated cells of 18 DABD.
(PDF)

S3 Fig Fluorescence microscopy of *P. putida* KT2440 cells treated with the kit “Live/Dead BacLight Bacterial Viability” from treatments desiccated without protectors. A) Bacterial cells before desiccation. B) Bacterial cells at 18 DABD. C) Bacterial cell adhered to germinated seeds. D) Rhizosphere colonization of *P*. *putida* KT2440 from plants inoculated with rehydrated cells of 18 DABD. Each row shows two captured imagens; the images of column SYTO 9 were taken at filter with excitation 420–490 nm, images of column propidium iodide were taken at filter excitation 500-550nm. MERGE correspond to combination of both images (SYTO 9 and propidium iodide).
(PDF)

S4 Fig MERGE Imagens and histograms that represent distribution of SYTO 9 and propidium iodide from cells random selected. Treatments desiccated without protectors. A) Bacterial cells before desiccation. B) Bacterial cells at 18 DABD. C) Bacterial cell adhered to germinated seeds. D) Rhizosphere colonization of *P*. *putida* KT2440 from plants inoculated with rehydrated cells of 18 DABD.
(PDF)

S5 Fig Fluorescence of *P. putida* KT2440 cells desiccated during 18 days, and stained with LIVE/DEAD BacLight Bacterial Viability kit. A) Before desiccation, B) 3, C) 6, D) 9, E) 12, F) 15, and G) 18 DABD. Each row shows two captured imagens; the images of column SYTO 9 were taken at filter with excitation 420–490 nm, images of column propidium iodide were taken at filter excitation 500-550nm. MERGE correspond to combination of both images (SYTO 9 and propidium iodide).
(TIFF)

S6 Fig MERGE imagens and histograms that represent distribution of SYTO 9 and propidium iodide from cells random selected. A) Before desiccation, B) 3, C) 6, D) 9, E) 12, F) 15, and G) 18 DABD
(PDF)

S7 Fig Fluorescence of *P*. *putida* KT2440 (A) before desiccation (B) after desiccation by 18 days and rehydrated by 20 minutes (C) 24 h (D) 48 h stained with LIVE/DEAD BacLight Bacterial Viability kit. The images of column SYTO 9 were taken at filter with excitation 420–490 nm, images of column propidium iodide were taken at filter excitation 500-550nm and images MERGE corresponding to combination of both images (SYTO 9 and propidium iodide).
(PDF)

S8 Fig MERGE imagens and histograms that represent distribution of SYTO 9 and propidium iodide from cells random selected. A) Before desiccation. B) Twenty-min rehydrated bacterial cells of 18 DABD. C) Twenty four-hour rehydrated bacterial cells of 18 DABD. D) Forty eight-hour rehydrated bacterial cells of 18 DABD.
(PDF)

S9 Fig Optical microscopy of *P. putida* KT2440 stained with toluidine blue along desiccation assay. A) Before desiccation, B) 6 DABD, C) 9 DABD, D) 12 DABD, E) 15 DABD, and F) 18 DABD.
(PDF)

S10 Fig Amplification of the16S rRNA gene from *P. putida* KT2440 by using the RT-PCR method. 1) Marker 1 kb DNA Leader Jena Bioscience, 2) cells before desiccation, 3) twenty-min rehydrated cells of 18 DABD. 4) Twenty four-hours rehydrated cells of 18 DABD, 5) twenty-min rehydrated cells of 40 DABD, 6) negative control; reaction without retrotranscriptase, and 7) negative control; reaction without template.
(PDF)

S11 Fig Amplification of the genes *mutL, rpoN* and *oprH* from *P. putida* KT2440 by using the RT-PCR method. 1) Marker 1 kb DNA Leader Jena Bioscience, 2) *mutL* before desiccation, 3) *mutL* from twenty-min rehydrated cells of 18 DABD, 4) *mutL* from Twenty four-hours rehydrated cells of 18 DABD, 5) *mutL* from twenty-min rehydrated cells of 40 DABD, 6) *rpoN* before desiccation, 7) *rpoN* from twenty-min rehydrated cells of 18 DABD, 8) *rpoN* from twenty four-hours rehydrated cells of 18 DABD, 9) *rpoN* from twenty-min rehydrated cells of 40 DABD, 10) *oprH* before desiccation, 11) *oprH* from twenty-min rehydrated cells of 18 DABD, 12) *oprH* from twenty four-hours rehydrated cells of 18 DABD, 13) *oprH* from twenty-min rehydrated cells of 40 DABD.
(PDF)
