# Supplementary material for: Desiccation-induced viable but nonculturable state in Pseudomonas putida KT2440, a survival strategy
Source: PLoS One. 2019 Jul 19;14(7):e0219554. doi: 10.1371/journal.pone.0219554 (PMC6641147; doi:10.1371/journal.pone.0219554)
Supplement: S3 Fig — A) Bacterial cells before desiccation. B) Bacterial cells at 18 DABD. C) Bacterial cell adhered to germinated seeds. D) Rhizosphere colonization of P. putida KT2440 from plants inoculated with rehydrated cells of 18 DABD. Each row shows two captured imagens; the images of column SYTO 9 were taken at filter with excitation 420–490 nm, images of column propidium iodide were taken at filter excitation 500-550nm. MERGE correspond to combination of both images (SYTO 9 and propidium iodide). (PDF) [file pone.0219554.s003.pdf]

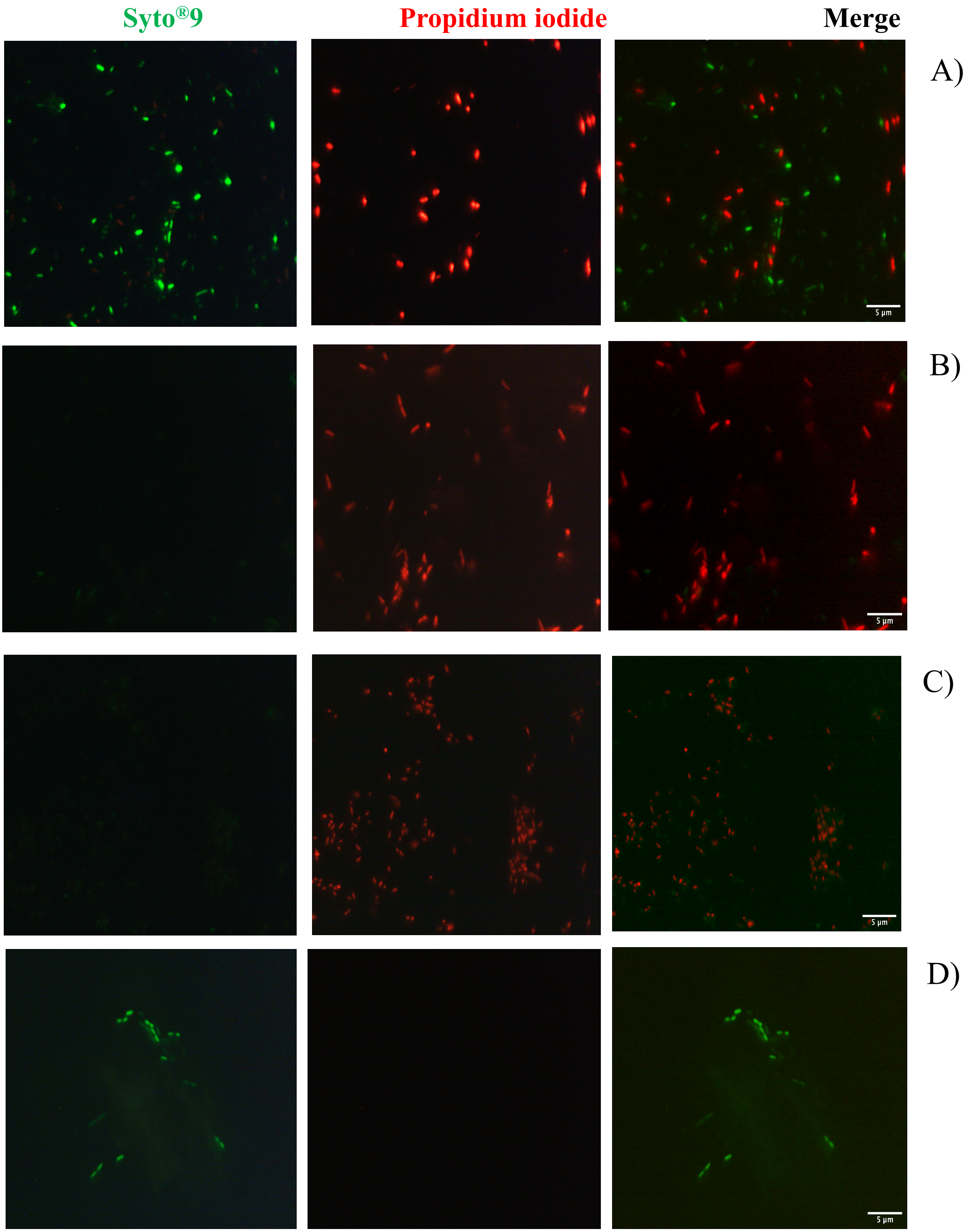

S3 Fig. Fluorescence microscopy of *P. putida* KT2440 cells treated with the kit "Live/Dead® BacLight™ Bacterial Viability" from treatments desiccated without protectors. A) Bacterial cells before desiccation. B) Bacterial cells at 18 DABD. C) Bacterial cell adhered to germinated seeds. D) Rhizosphere colonization of *P. putida* KT2440 from plants inoculated with rehydrated cells of 18 DABD. Each row shows two captured imagens; the images of column SYTO®9 were taken at filter with excitation 420-490 nm, images of column propidium iodide were taken at filter excitation 500-550nm. MERGE correspond to combination of both images (SYTO®9 and propidium iodide).
