# Supplementary material for: Desiccation-induced viable but nonculturable state in Pseudomonas putida KT2440, a survival strategy
Source: PLoS One. 2019 Jul 19;14(7):e0219554. doi: 10.1371/journal.pone.0219554 (PMC6641147; doi:10.1371/journal.pone.0219554)
Supplement: S4 Fig — Treatments desiccated without protectors. A) Bacterial cells before desiccation. B) Bacterial cells at 18 DABD. C) Bacterial cell adhered to germinated seeds. D) Rhizosphere colonization of P. putida KT2440 from plants inoculated with rehydrated cells of 18 DABD. (PDF) [file pone.0219554.s004.pdf]

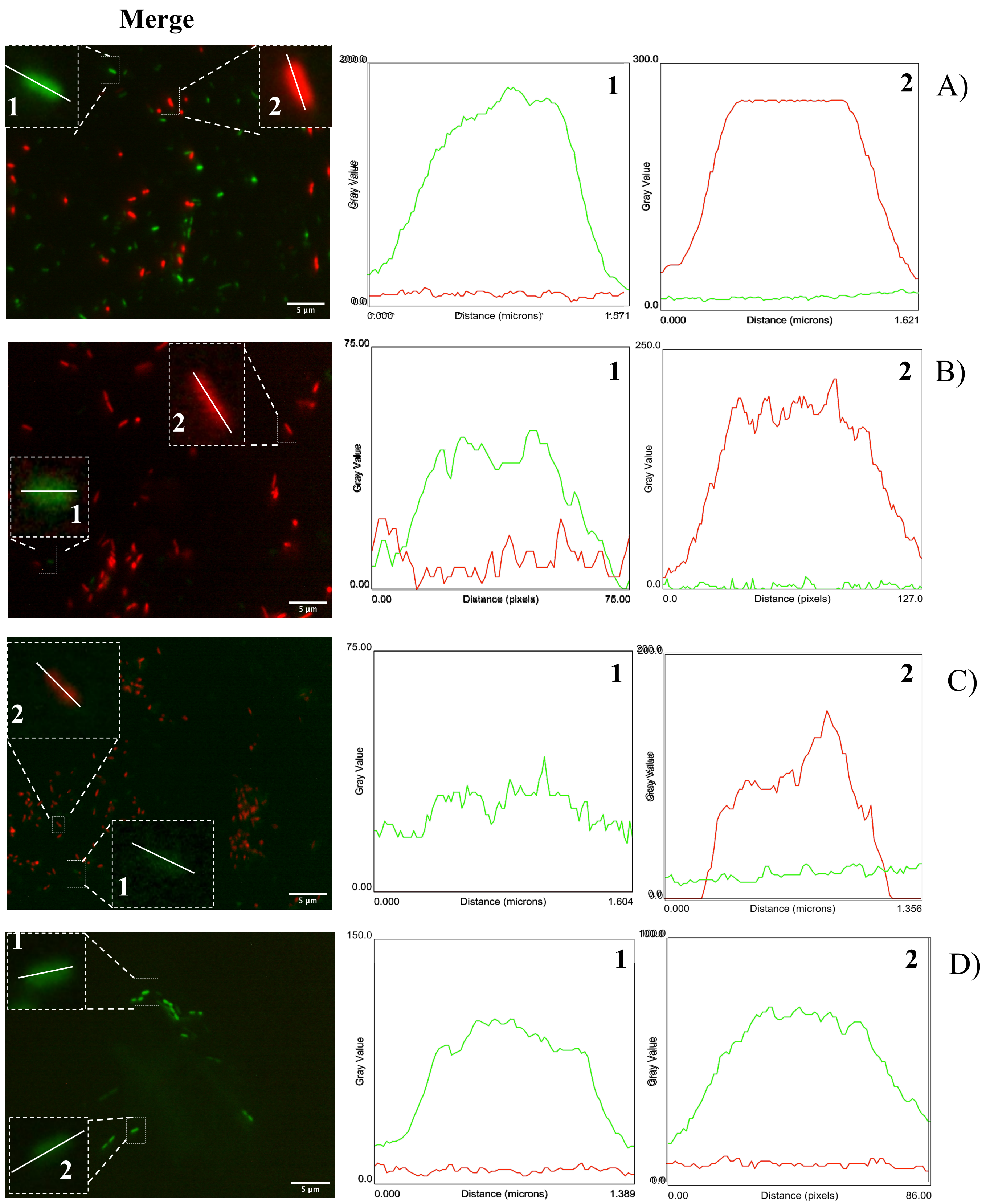

S4 Fig. MERGE Imagens and histograms that represent distribution of SYTO®9 and propidium iodide from cells random selected. Treatments desiccated without protectors. A) Bacterial cells before desiccation. B) Bacterial cells at 18 DABD. C) Bacterial cell adhered to germinated seeds. D) Rhizosphere colonization of *P. putida* KT2440 from plants inoculated with rehydrated cells of 18 DABD.
