# Supplementary figures and images for: Desiccation-induced viable but nonculturable state in Pseudomonas putida KT2440, a survival strategy
Source: PLoS One. 2019 Jul 19;14(7):e0219554. doi: 10.1371/journal.pone.0219554 (PMC6641147; doi:10.1371/journal.pone.0219554)

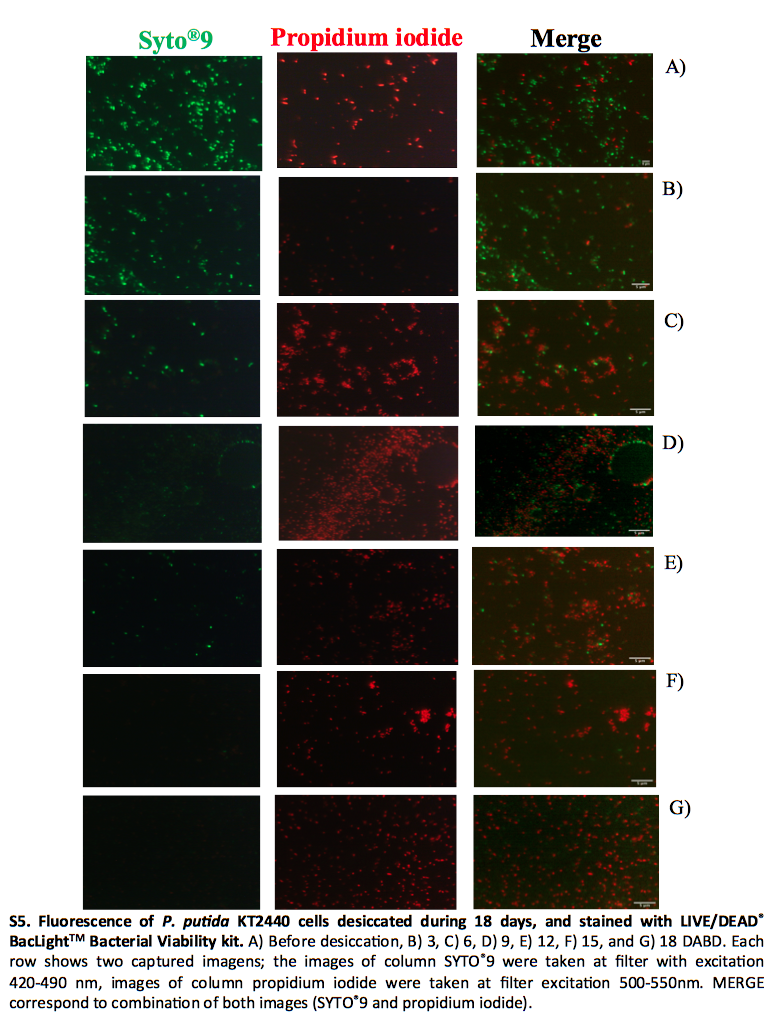

Supplement: S5 Fig — A) Before desiccation, B) 3, C) 6, D) 9, E) 12, F) 15, and G) 18 DABD. Each row shows two captured imagens; the images of column SYTO 9 were taken at filter with excitation 420–490 nm, images of column propidium iodide were taken at filter excitation 500-550nm. MERGE correspond to combination of both images (SYTO 9 and propidium iodide). (TIFF) [file pone.0219554.s005.tiff]
