# Supplementary material for: Desiccation-induced viable but nonculturable state in Pseudomonas putida KT2440, a survival strategy
Source: PLoS One. 2019 Jul 19;14(7):e0219554. doi: 10.1371/journal.pone.0219554 (PMC6641147; doi:10.1371/journal.pone.0219554)
Supplement: S6 Fig — A) Before desiccation, B) 3, C) 6, D) 9, E) 12, F) 15, and G) 18 DABD (PDF) [file pone.0219554.s006.pdf]

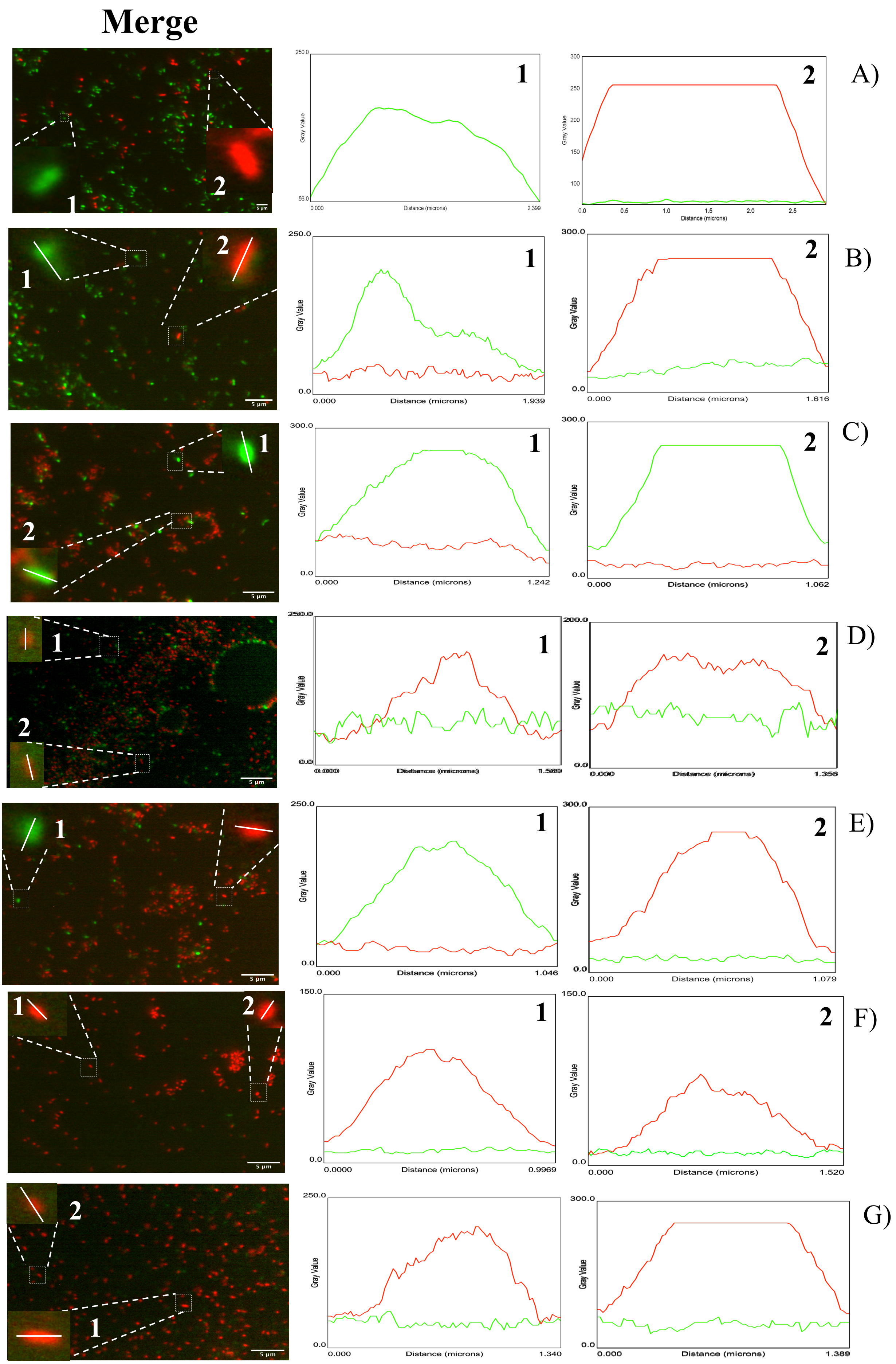

Merge
A)
B)
C)
D)
E)
F)
G)
1
2
Gray Value
Distance (microns)
250.0
56.0
0.000
2.399
300
250
200
150
100
0.0
0.5
1.0
1.5
2.0
2.5
0.0
1.939
300.0
1.616
1.242
1.062
200.0
1.569
1.356
1.046
1.079
150.0
0.0000
0.9969
1.520
1.340
1.389
5 µm

**S6 Fig. MERGE imagens and histograms that represent distribution of SYTO®9 and propidium iodide from cells random selected**. A) Before desiccation, B) 3, C) 6, D) 9, E) 12, F) 15, and G) 18 DABD.
