# Supplementary material for: Desiccation-induced viable but nonculturable state in Pseudomonas putida KT2440, a survival strategy
Source: PLoS One. 2019 Jul 19;14(7):e0219554. doi: 10.1371/journal.pone.0219554 (PMC6641147; doi:10.1371/journal.pone.0219554)
Supplement: S7 Fig — Fluorescence of P. putida KT2440 (A) before desiccation (B) after desiccation by 18 days and rehydrated by 20 minutes (C) 24 h (D) 48 h stained with LIVE/DEAD BacLight Bacterial Viability kit. The images of column SYTO 9 were taken at filter with excitation 420–490 nm, images of column propidium iodide were taken at filter excitation 500-550nm and images MERGE corresponding to combination of both images (SYTO 9 and propidium iodide). (PDF) [file pone.0219554.s007.pdf]

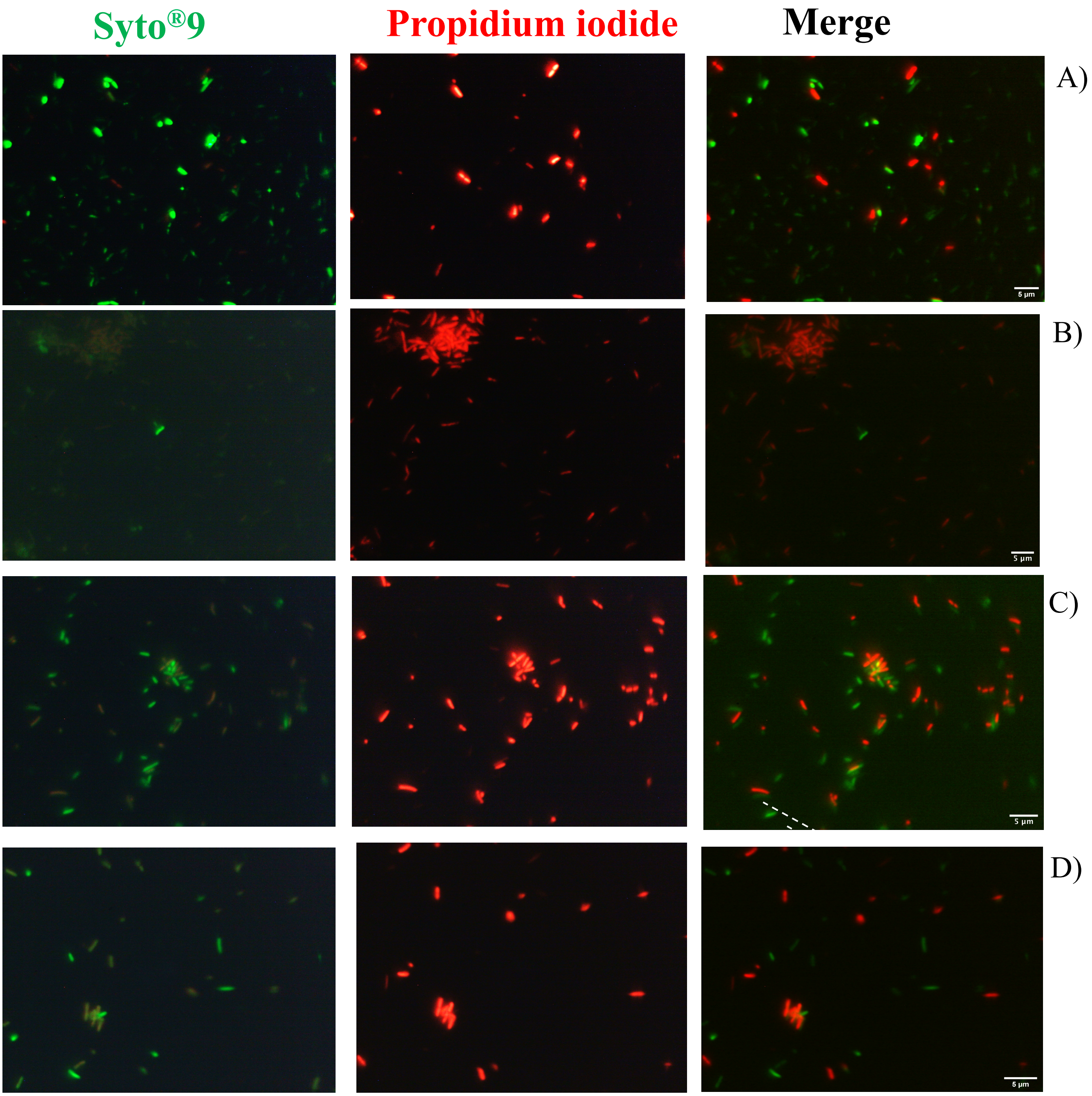

S7 Fig. Fluorescence of *P. putida* KT2440 (A) before desiccation (B) after desiccation by 18 days and rehydrated by 20 minutes (C) 24 h (D) 48 h stained with LIVE/DEAD® BacLight™ Bacterial Viability kit. The images of column SYTO®9 were taken at filter with excitation 420-490 nm, images of column propidium iodide were taken at filter excitation 500-550nm and images MERGE corresponding to combination of both images (SYTO®9 and propidium iodide).
