# Supplementary material for: Desiccation-induced viable but nonculturable state in Pseudomonas putida KT2440, a survival strategy
Source: PLoS One. 2019 Jul 19;14(7):e0219554. doi: 10.1371/journal.pone.0219554 (PMC6641147; doi:10.1371/journal.pone.0219554)
Supplement: S8 Fig — A) Before desiccation. B) Twenty-min rehydrated bacterial cells of 18 DABD. C) Twenty four-hour rehydrated bacterial cells of 18 DABD. D) Forty eight-hour rehydrated bacterial cells of 18 DABD. (PDF) [file pone.0219554.s008.pdf]

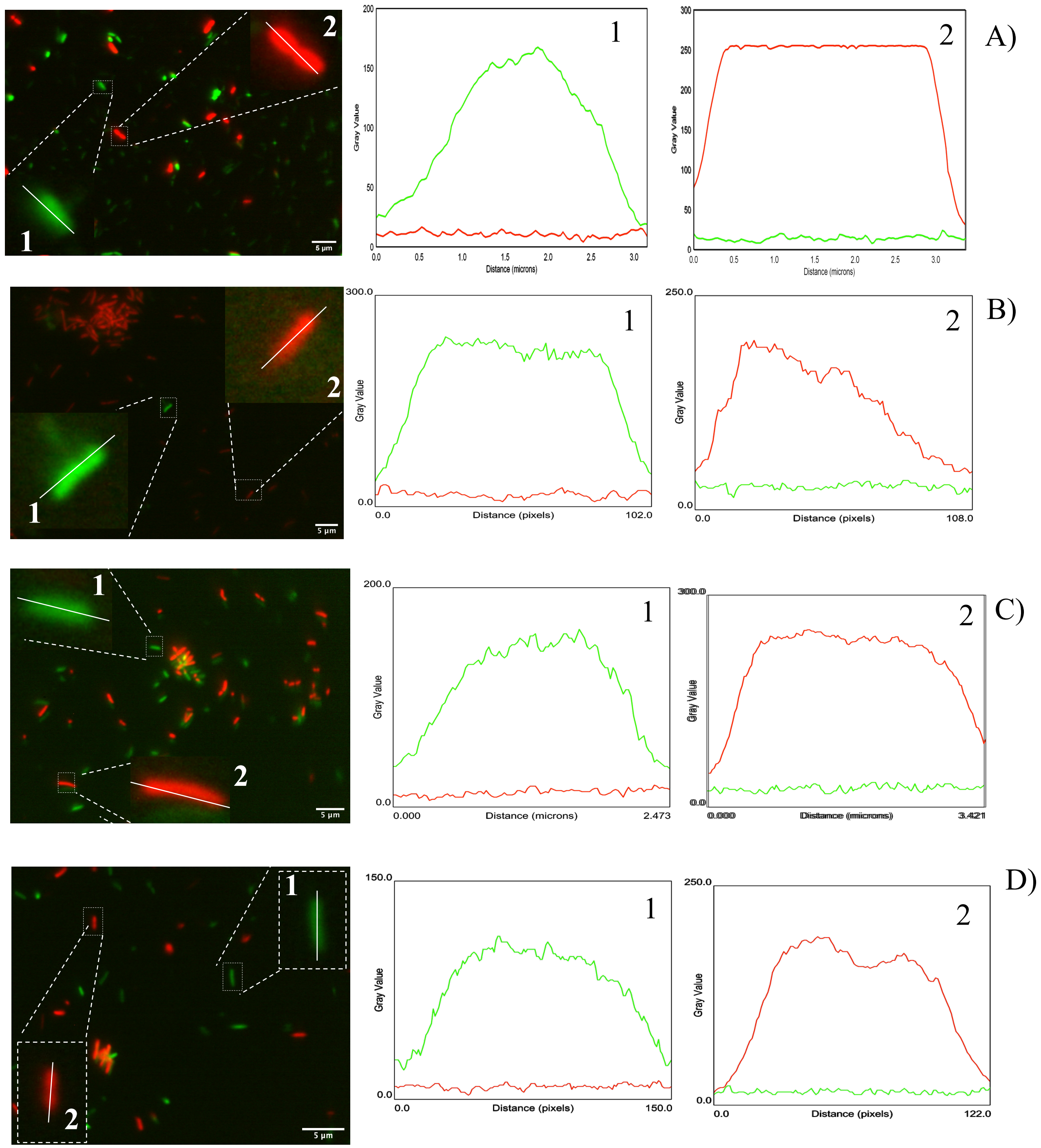

S8 Fig. MERGE imagens and histograms that represent distribution of SYTO®9 and propidium iodide from cells random selected. A) Before desiccation. B) Twenty-min rehydrated bacterial cells of 18 DABD. C) Twenty four-hour rehydrated bacterial cells of 18 DABD. D) Forty eight-hour rehydrated bacterial cells of 18 DABD.
