# Supplementary material for: Desiccation-induced viable but nonculturable state in Pseudomonas putida KT2440, a survival strategy
Source: PLoS One. 2019 Jul 19;14(7):e0219554. doi: 10.1371/journal.pone.0219554 (PMC6641147; doi:10.1371/journal.pone.0219554)
Supplement: S9 Fig — A) Before desiccation, B) 6 DABD, C) 9 DABD, D) 12 DABD, E) 15 DABD, and F) 18 DABD. (PDF) [file pone.0219554.s009.pdf]

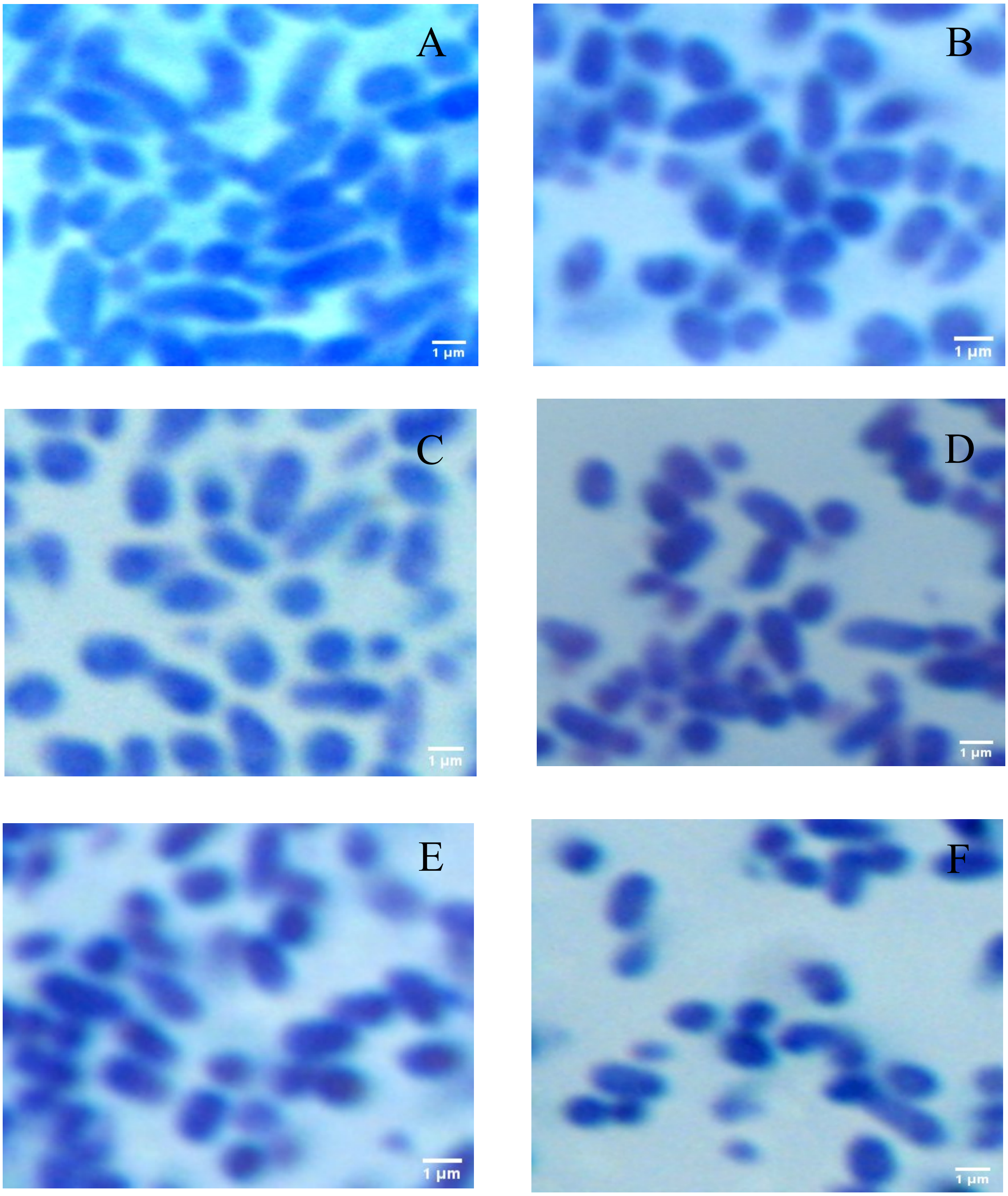

S9 Fig. Optical microscopy of *P. putida* KT2440 stained with toluidine blue along desiccation assay. A) Before desiccation, B) 6 DABD, C) 9 DABD, D) 12 DABD, E) 15 DABD, and F) 18 DABD.
