# Supplementary material for: Desiccation-induced viable but nonculturable state in Pseudomonas putida KT2440, a survival strategy
Source: PLoS One. 2019 Jul 19;14(7):e0219554. doi: 10.1371/journal.pone.0219554 (PMC6641147; doi:10.1371/journal.pone.0219554)
Supplement: S10 Fig — 1) Marker 1 kb DNA Leader Jena Bioscience, 2) cells before desiccation, 3) twenty-min rehydrated cells of 18 DABD. 4) Twenty four-hours rehydrated cells of 18 DABD, 5) twenty-min rehydrated cells of 40 DABD, 6) negative control; reaction without retrotranscriptase, and 7) negative control; reaction without template. (PDF) [file pone.0219554.s010.pdf]

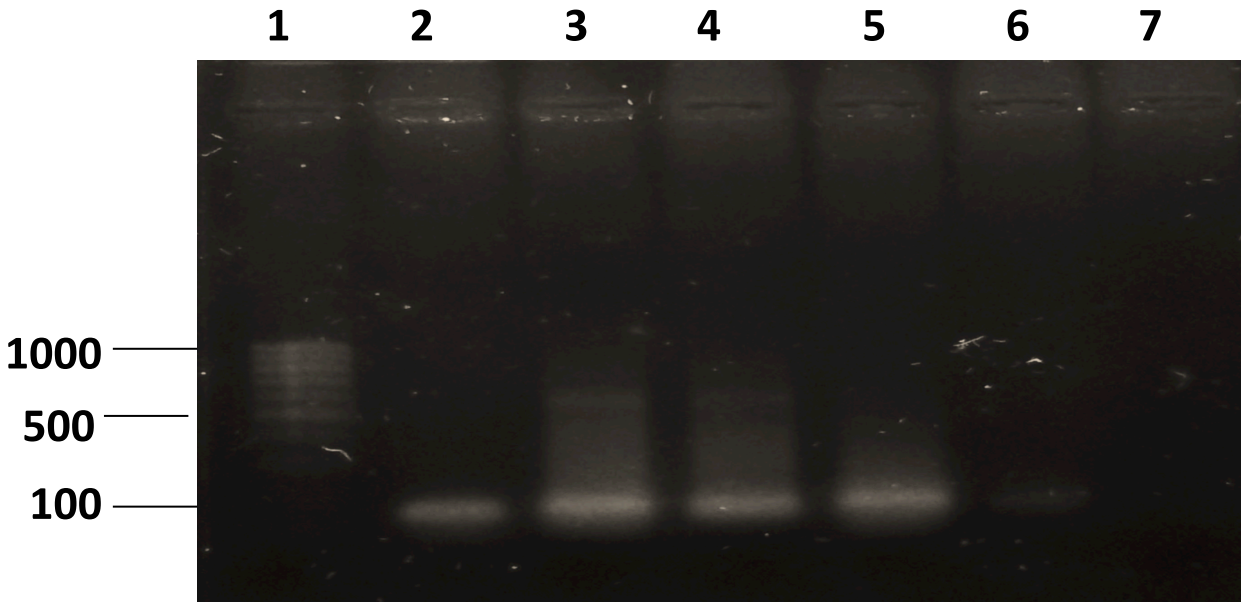

S10 Fig. Amplification of the16S rRNA gene from *P. putida* KT2440 by using the RT-PCR method. 1) Marker 1 kb DNA Leader Jena Bioscience, 2) cells before desiccation, 3) twenty-min rehydrated cells of 18 DABD. 4) Twenty four-hours rehydrated cells of 18 DABD, 5) twenty-min rehydrated cells of 40 DABD, 6) negative control; reaction without retrotranscriptase, and 7) negative control; reaction without template.
