# Supplementary material for: Desiccation-induced viable but nonculturable state in Pseudomonas putida KT2440, a survival strategy
Source: PLoS One. 2019 Jul 19;14(7):e0219554. doi: 10.1371/journal.pone.0219554 (PMC6641147; doi:10.1371/journal.pone.0219554)
Supplement: S11 Fig — 1) Marker 1 kb DNA Leader Jena Bioscience, 2) mutL before desiccation, 3) mutL from twenty-min rehydrated cells of 18 DABD, 4) mutL from Twenty four-hours rehydrated cells of 18 DABD, 5) mutL from twenty-min rehydrated cells of 40 DABD, 6) rpoN before desiccation, 7) rpoN from twenty-min rehydrated cells of 18 DABD, 8) rpoN from twenty four-hours rehydrated cells of 18 DABD, 9) rpoN from twenty-min rehydrated cells of 40 DABD, 10) oprH before desiccation, 11) oprH from twenty-min rehydrated cells of 18 DABD, 12) oprH from twenty four-hours rehydrated cells of 18 DABD, 13) oprH from twenty-min rehydrated cells of 40 DABD. (PDF) [file pone.0219554.s011.pdf]

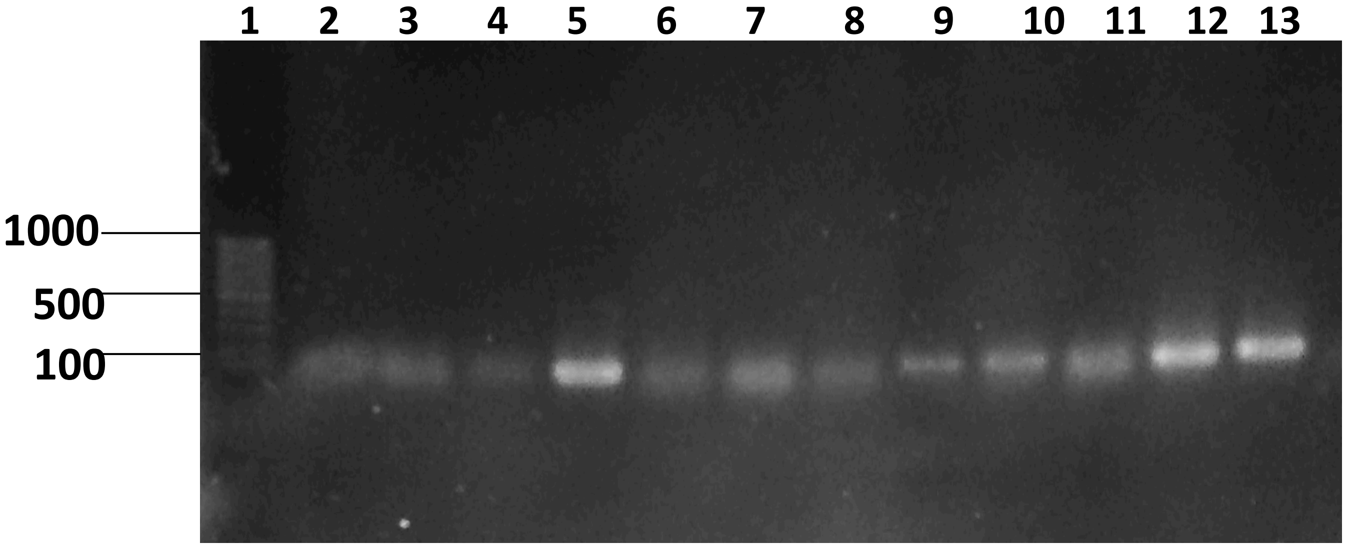

S11 Fig. Amplification of the genes *mutL, rpoN* and *oprH* from *P. putida* KT2440 by using the RT-PCR method. 1) Marker 1 kb DNA Leader Jena Bioscience, 2) *mutL* before desiccation, 3) *mutL* from twenty-min rehydrated cells of 18 DABD, 4) *mutL* from Twenty four-hours rehydrated cells of 18 DABD, 5) *mutL* from twenty-min rehydrated cells of 40 DABD, 6) *rpoN* before desiccation, 7) *rpoN* from twenty-min rehydrated cells of 18 DABD, 8) *rpoN* from twenty four-hours rehydrated cells of 18 DABD, 9) *rpoN* from twenty-min rehydrated cells of 40 DABD, 10) *oprH* before desiccation, 11) *oprH* from twenty-min rehydrated cells of 18 DABD, 12) *oprH* from twenty four-hours rehydrated cells of 18 DABD, 13) *oprH* from twenty-min rehydrated cells of 40 DABD.
